# A weighted taxonomic matrix key for species of the rotifer genus *Synchaeta* (Rotifera, Monogononta, Synchaetidae)

**DOI:** 10.3897/zookeys.871.36435

**Published:** 2019-08-12

**Authors:** Tanja Wilke, Wilko H. Ahlrichs, Olaf R.P. Bininda-Emonds

**Affiliations:** 1 AG Systematik und Evolutionsbiologie, Institut für Biologie und Umweltwissenschaften (IBU), Carl von Ossietzky Universität Oldenburg, Carl-von-Ossietzky Straße 9–11, 26111 Oldenburg, Germany Carl von Ossietzky Universität Oldenburg Oldenburg Germany

**Keywords:** Habitus, morphology, robust characters, species identification, swimming behaviour, trophi

## Abstract

A new, weighted matrix identification key for 34 largely undisputed species of *Synchaeta* was created with the aim of providing comparable, detailed and diagnostic character sets for each species that can be applied to live and/or preserved specimens. As part of this process, 14 species of *Synchaeta* were intensively re-investigated with respect to their habitus and trophi morphology using binocular, light, and scanning electron microscopy, which, together with behavioural observations, revealed several new discriminating characters. Whenever possible, missing information for any character was added for the remaining species from the literature, with the two recently described species *Synchaeta arcifera* and *Synchaeta squamadigitata* being considered for the first time in an identification key. Beyond its completeness, our key has two distinct advantages. First, the characters are supported by detailed illustrations of their respective character states whenever possible to both simplify identification and minimize any uncertainty in the descriptions themselves. Second, the new approach of weighting the characters according to their reliability, robustness and/or ease of determination was employed. This latter approach is especially advantageous for soft-bodied rotifers such as species of *Synchaeta*, where, for example, several external characters can be influenced by preservation and are therefore less diagnostic or reliable. Although the key is as comprehensive as possible, information for many species remains missing for many characters, thereby highlighting the need for additional comprehensive and detailed species (re-)investigations within *Synchaeta*.

## Introduction

The rotifer genus *Synchaeta* (Monogononta, Synchaetidae) comprises approximately 37 (see Segers 2007) to 39 (see Jersabek et al. 2018) valid and truly planktonic species, of which approximately half occur in brackish and/or marine habitats (Hollowday 2002). Although their importance in aquatic food webs is unquestioned because of their often dominant role in the rotifer (Stemberger and Gilbert 1985) and metazooplankton communities (Arndt et al. 1990), specimens of *Synchaeta* in ecological studies are seldom identified to species level (Obertegger et al. 2006). To a large extent, this situation derives from the identification of and delimitation between species of this genus being regarded as being especially challenging (Pourriot 1965; Ruttner-Kolisko 1972; Koste 1978).

The several comprehensive revisions and keys of *Synchaeta* that exist (e.g., Voigt 1956–1957; Ruttner-Kolisko 1972; Koste 1978; and most recently Hollowday 2002) tend to be restricted in that they limit themselves to describing the most concise set of characters that delimit each species. Although this represents a useful simplifying strategy, the inherently incomplete data set it entails presents two distinct disadvantages. First, because additional, alternative characters are not presented for many species, their identification is impossible when the respective, diagnostic ones are deformed or not clearly visible (e.g., foot morphology when it is retracted). Second, and more importantly, direct comparisons among species are usually not possible because the species are often described using different sets of characters.

To address both sets of issues, we have developed a new identification key for *Synchaeta*, with the dual aims of making it both easy to use and as comprehensive as possible by providing large, comparable data sets for each species. To accomplish this, we thoroughly re-examined live and prepared specimens of 14 species and intensively researched the literature for all members of *Synchaeta*, including the most recently described species *Synchaeta arcifera* Xu, 1998 and *Synchaeta squamadigitata* De Smet, 2006, which are presented for the first time in a comparative identification key. In addition to an in-depth analysis of the habitus, we focussed on the trophi in particular because they are considered to be both species-specific (De Smet 1998; Fontaneto and Melone 2006; Wulfken et al. 2010) and less susceptible to conservation (Kutikova 1970 as cited in Obertegger et al. 2006). Detailed information about the trophi are therefore of great advantage in ecological studies, for example, where the material is necessarily fixed for practical reasons (Obertegger et al. 2006; Labuce and Strake 2017), with the consequence that the species identity of distorted or contracted rotifer specimens might be determinable only via their trophi (De Smet 1998; Segers 2004). By providing comparable data sets for each species, our taxonomic key also functions as a matrix key (also known as free access or multi-access key; see Hagedorn et al. 2010), which is better able to incorporate missing information when identifying species than the more traditional dichotomous key.

In addition, we weighted all characters within our matrix key according to their perceived discriminatory power. This strategy of focussing on more robust and diagnostically conclusive characters potentially facilitates accurate species identification by giving characters that are more susceptible to variation or artefacts (e.g., body shape, which is strongly affected by the pressure of a cover slip (Koste 1978), fixation/preservation (Ruttner-Kolisko 1972; Segers 2004) or by developing eggs and stomach content in soft-bodied rotifers) less impact than more constant and robust ones (e.g., the number and position of the lateral antennae.) To further simplify the identification process, we supported the characters with detailed illustrations and photographs of the respective character states whenever possible and introduce a consistent and distinctive terminology for homologous structures. Although the latter point seems obvious, the use of synonyms for homologous structures is a widespread problem, even within the same key. For example, Hollowday (2002: 90) variously denotes the apical receptors as “sensory frontal antennae”, “sensory antenna”, “frontal prominence with tuft of setae” or “sensory setae” in his identification key for species of *Synchaeta*.

Our purpose here is to deliver a comprehensive and robust key for *Synchaeta* by which a reliable identification of live and preserved specimens is feasible through a comprehensive and comparable morphological data set. In so doing, the present study not only confirmed existing discriminatory characters, but also re-described some of them more explicitly (e.g., foot shape and morphology of the apical receptors) as well as established several novel ones for species demarcation (as e.g., behaviour, morphology of the pedal glands, detailed fulcrum and ramus morphology).

## Materials and methods

### Study site and sampling

Using a 55-µm mesh plankton net, sampling for species of *Synchaeta* took place intermittently between June 2013 and August 2017 in northwest Germany from freshwater habitats in Oldenburg, Lower Saxony and Tecklenburger Land, North Rhine-Westphalia as well as from brackish and marine habitats in Wilhelmshaven, Lower Saxony (Table 1). Species of *Synchaeta* found in the samples (Table 2) were identified using the existing information in Rousselet (1902), Voigt (1956–1957), Ruttner-Kolisko (1972), Koste (1978) and Hollowday (2002).

### Binocular and light microscopical (LM) investigations

Undisturbed, living specimens were initially observed in a petri dish using a binocular microscope to examine their (swimming) behaviour. For the LM analyses, single individuals were isolated and carefully sedated with carbonated water before being further immobilized through the slight pressure of a cover glass. For the latter step, extreme care was taken to avoid any deformation of the body, which could lead to morphological artefacts. All observations used differential interference contrast using a LEICA DMLB microscope and digital photographs were taken using a Canon EOS 5D Mark II camera.

**Table 1.** Sampling locations with their corresponding coordinates and habitat characterizations. Abbreviations: OL = Oldenburg, TL = Tecklenburger Land, WHV = Wilhelmshaven.

Location		Coordinates	Type of Habitat	Salinity in PSU
Schlossteich (ST)	OL	53.1603N; 8.1195E	permanent freshwater pond	0
Löschteich (LT)	OL	53.151957N; 8.166833E	permanent freshwater pond	0
Haarenniederung (HN)	OL	53.147092N; 8.171273E	temporary freshwater winter puddle	0
Haarenstau (HS)	OL	53.155623N; 8.105789E	temporary freshwater winter puddle	0
Heiliges Meer (HM)	TL	52.351944N; 7.633611E	permanent freshwater lake	0
Banter See (BS)	WHV	53.50906N; 8.1143116E	tide-independent, brackish basin	ca. 8
Yachthafen (YH)	WHV	53.5097712N; 8.1216346E	tide-independent, brackish basin	ca. 20
Nassauhafen (NH)	WHV	53.5129901N; 8.1458015E	North Sea coast, marine habitat	ca. 30

**Table 2.** Sampled species of *Synchaeta*. Abbreviations: BS = Banter See; HM = Heiliges Meer; HN = Haarenniederung; HS = Haarenstau; LT = Löschteich; NH = Nassauhafen; ST = Schlossteich; YH = Yachthafen.

Species	Location	Date	Number of specimens examined
**From freshwater habitats**			
*S. grandis*	ST	June 2016	18
*S. kitina*	HM	April 2017	ca. 25
*S. longipes*	ST	June and July 2013	15
*S. oblonga*	ST	March 2016	ca. 70
*S. pectinata*	ST	April to June 2013 and 2015	ca. 120
*S. stylata*	ST	June to August 2016 and 2017	ca. 50
*S. tremula*	ST, LT	March to May 2016	ca. 90
*S. tremuloida*	HS, HN	November to January 2015/2016	ca. 70
**From brackish and marine habitats**			
*S. triophthalma*	NH	April 2016	ca. 25
*S. hutchingsi*	YH	August 2017	18
*S. grimpei*	YH, NH	April 2016	13
*S. gyrina*	BS, NH	January and April 2016	ca. 60
*S. baltica*	BS, NH	January and April 2016	ca. 35
*S. vorax*	NH	April 2016	17

### Scanning electron microscopical (SEM) investigations

For SEM examinations of the habitus, single specimens were initially sedated with carbonated water before being euthanized with 1% OsO_4_ buffered in 0.1 M NaCa cacodylate buffer and fixed with 240 mOsmol picric acid-formaldehyde (Melone and Ricci 1995). To examine the isolated trophi, the surrounding tissue of selected specimens was dissolved according to the protocol of Kleinow et al. (1990) by transferring them into a droplet of dissolving agent (0.1 g dithiothreitol added to a 5-ml stock solution of 5.2 g sodium dodecyl sulphate + 0.24 g NH_4_HCO_3_ in 100 ml aqua dest; AppliChem, Darmstadt, Germany) for ca. 15 min before being rinsed with distilled water subsequently. Thereafter, samples of either the habitus or the trophi were dehydrated using an ascending, graded ethanol series. Following critical-point drying, each sample was attached onto an SEM stub and coated with gold-palladium before being examined on a Hitachi S-3200N SEM.

### Illustrations

All new illustrations of the habitus were made using Adobe Illustrator CS4 based on representative digital photographs. References from drawings that we have obtained and modified from literature are listed below each illustration.

### Included species and information from the literature

Overall, 34 species of *Synchaeta* were considered in our key, with six species that are recognized by either Segers (2007) and/or Jersabek et al. (2018) being excluded (see lists below). Information about species that we did not find in our samples derive from their respective initial descriptions and from the literature, with an emphasis on Rousselet (1902), Lauterborn (1905), Lie-Pettersen (1905), Peters (1931), Voigt (1956–1957), Ruttner-Kolisko (1972), Koste (1978) and Hollowday (2002). Information or interpretations that we have made from illustrations or photographs that are derived from other sources than the above-mentioned literature are indicated below each table (Tables 3–8). Species that are in urgent need of re-investigation because of inconsistent, ambiguous or highly incomplete descriptions (see Tables 3–8; “?“) and/or species that are known exclusively from preserved material are indicated with an asterisk in the following lists.

### List of recognized freshwater species (Fig. 1A–M):

**Figure 1.**
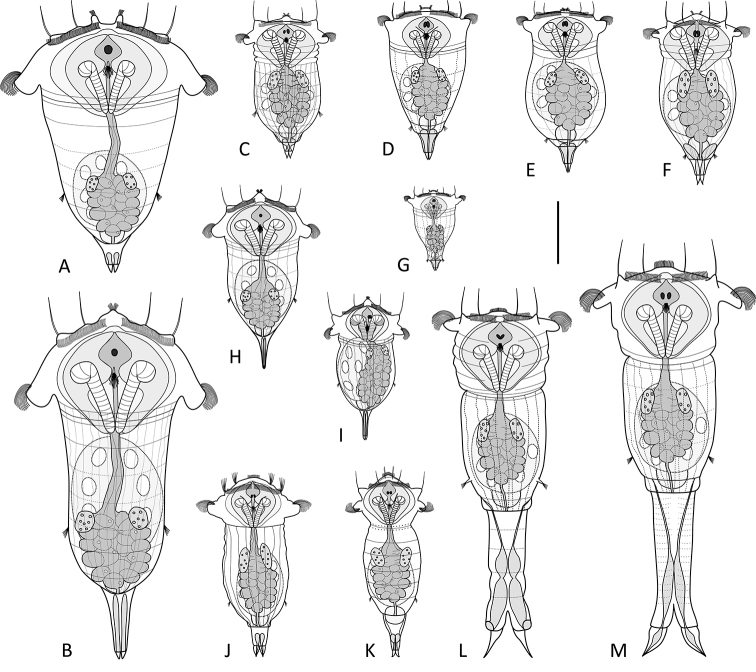
Species of *Synchaeta* from freshwater habitats. **A***S. pectinata***B***S. grandis***C***S. oblonga***D***S. tremula***E***S. tremuloida***F***S. prominula***G***S. kitina***H***S. stylata***I***S. longipes***J***S. verrucosa***K***S. lakowitziana***L***S. pachypoida***M***S. pachypoda*. Drawings modified from: **F, L, M**Kutikova and Vassiljeva (1982)**J**Jersabek et al. (2003b)**K**Lucks (1930). Scale bar: 100 µm.

*Synchaeta pectinata* Ehrenberg, 1832 (Fig. 1A)

*Synchaeta grandis* Zacharias, 1893 (Fig. 1B)

*Synchaeta oblonga* Ehrenberg, 1832 (Fig. 1C)

*Synchaeta tremula* (Müller, 1786) (Fig. 1D)

*Synchaeta tremuloida* Pourriot, 1965 (Fig. 1E)

*Synchaeta prominula* Kutikova & Vassiljeva, 1982 (Fig. 1F)*

*Synchaeta kitina* Rousselet, 1902 (Fig. 1G)

*Synchaeta stylata* Wierzejski, 1893 (Fig. 1H)

*Synchaeta longipes* Gosse, 1887 (Fig. 1I)

*Synchaeta verrucosa* Nipkow, 1961 (Fig. 1J)

*Synchaeta lakowitziana* Lucks, 1930 (Fig. 1K)*

*Synchaeta pachypoida* Kutikova & Vassiljeva, 1982 (Fig. 1L)*

*Synchaeta pachypoda* Jashnov, 1922 (Fig. 1M)*

### List of recognized brackish, marine or inland saline species (Fig. 2A–U):

**Figure 2.**
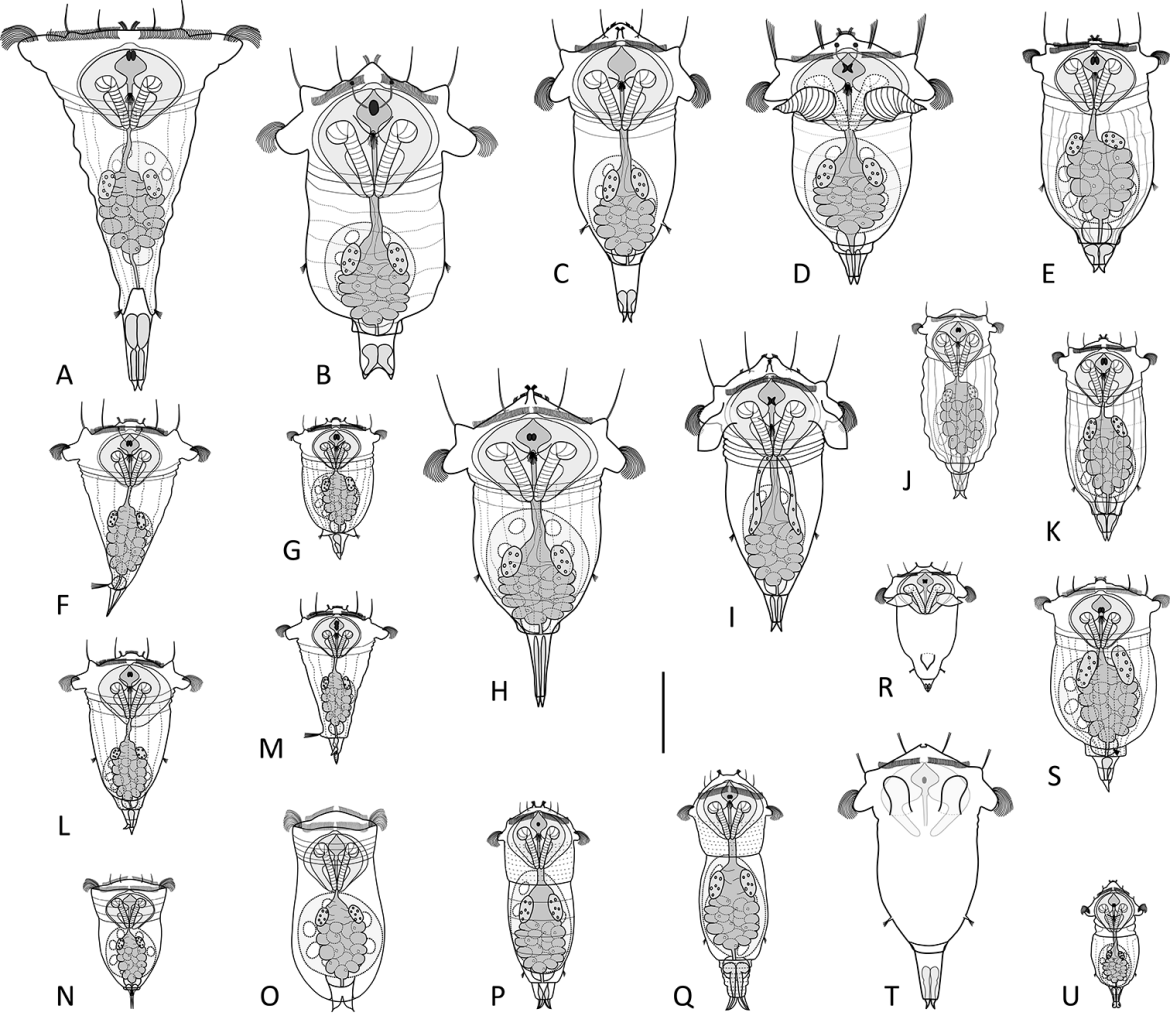
Species of *Synchaeta* from brackish, marine and inland saline habitats. **A***S. grimpei***B***S. baltica***C***S. johanseni***D***S. bicornis***E***S. gyrina***F***S. triophthalma***G***S. cecilia***H***S. vorax***I***S. fennica***J***S. cylindrica***K***S. tavina***L***S. neapolitana***M***S. hutchingsi***N***S. atlantica***O***S. rousseleti***P***S. glacialis***Q***S. hyperborea***R***S. arcifera***S***S. tamara***T***S. bacillifera***U***S. squamadigitata*. Drawings modified from: **C**Harring (1921)**D**Koste (1981)**G, K, L**Rousselet (1902)**I**Rousselet (1909)**J**Althaus (1957)**N**Zelinka (1907)**O**Zelinka (1927)**P, Q, S**Smirnov (1932) and Friedrich and De Smet (2000)**R**Rougier and Pourriot (2006)**T**Smirnov (1933)**U**De Smet (2006). Scale bar: 100 µm.

*Synchaeta grimpei* Remane, 1929 (Fig. 2A)

*Synchaeta baltica* Ehrenberg, 1834 (Fig. 2B)

*Synchaeta johanseni* Harring, 1921 (Fig. 2C)*

*Synchaeta bicornis* Smith, 1904 (Fig. 2D)*

*Synchaeta gyrina* Hood, 1887 (Fig. 2E)

*Synchaeta triophthalma* Lauterborn, 1894 (Fig. 2F)

*Synchaeta cecilia* Rousselet, 1902 (Fig. 2G)

*Synchaeta vorax* Rousselet, 1902 (Fig. 2H)

*Synchaeta fennica* Rousselet, 1909 (Fig. 2I)*

*Synchaeta cylindrica* Althaus, 1957 (Fig. 2J)*

*Synchaeta tavina* Hood, 1893 (Fig. 2K)*

*Synchaeta neapolitana* Rousselet, 1902 (Fig. 2L)*

*Synchaeta hutchingsi* Brownell, 1988 (Fig. 2M)

*Synchaeta atlantica* Zelinka, 1907 (Fig. 2N)*

*Synchaeta rousseleti* Zelinka, 1927 (Fig. 2O)*

*Synchaeta glacialis* Smirnov, 1932 (Fig. 2P)*

*Synchaeta hyperborea* Smirnov, 1932 (Fig. 2Q)*

*Synchaeta arcifera* Xu, 1998 (Fig. 2R)*

*Synchaeta tamara* Smirnov, 1932 (Fig. 2S)*

*Synchaeta bacillifera* Smirnov, 1933 (Fig. 2T)*

*Synchaeta squamadigitata* De Smet, 2006 (Fig. 2U)*

### Excluded species

In general, we excluded species of *Synchaeta* that are ranked as a species inquirenda (= species of doubtful identity) by Jersabek et al. (2018) or for which we strongly suspect this to be the case based on our own observations.

1. *Synchaeta curvata* Lie-Pettersen, 1905: Insufficient description (Hollowday 2002) and currently ranked as a species inquirenda in Segers (2007).

2. *Synchaeta elsteri* Hauer, 1963: Insufficient description based on preserved specimens (Hollowday 2002). This species was also ranked as a species inquirenda in Segers (2007).

3. *Synchaeta jollyae* Shiel & Koste, 1993: Described based on preserved specimens and synonymy with *S. stylata* suspected (Wilke et al. 2018a).

4. *Synchaeta littoralis* Rousselet, 1902: Synonymy with *S. oblonga* suspected (Koste 1978; Hollowday 2002; Wilke et al. 2018b). This species was also ranked as a species inquirenda in Segers (2007).

5. *Synchaeta monopus* Plate, 1889: Existing descriptions are insufficient, inconsistent and made exclusively on the basis of preserved and presumably deformed specimens (Wilke et al. 2019).

6. *Synchaeta rufina* Kutikova & Vassiljeva, 1982: Synonymy with *S. oblonga* suspected (Wilke et al. 2018b).

### Character clarification, character weighting, and species identification

To support the written descriptions, the morphology for each character state is also exemplified both through illustrations as well as the naming of at least one exemplar species that possesses the respective state.

The character states are represented in detailed tables (Tables 3–8) and in a numerical list for each species where the text is formatted according to the perceived reliability and/or discriminatory power of the states:

1. “?”: The character state is unknown or ambiguous for the respective species – further examinations are required.

2. brackets: The character state rarely occurs in the species.

3. *italics*: The character is variably expressed within the species or its interpretation is either subjective or can be easily misunderstood because of potential artefacts that can arise during preparation. These characters should be applied with caution.

4. normal text: The character state is more or less robust, but shared by several, additional species of *Synchaeta*. Many characters of this quality are usually required for species demarcation in the form of a unique character set for each species.

5. blue color: The character state is robust and important insofar as it is unique for the species and/or shared by only a few, additional species of *Synchaeta*. Individual characters in this category typically exclude many other congeneric species to greatly simplify species demarcation.

6. **bold**: The character state is robust and species-specific (autapomorphy).

To simplify the identification process, blank character checklists and tables for recording character states are appended (Suppl. material 1: Tables S1, S2).

## Results

The characters are categorized into those for habitat and behaviour (characters 1–6), size (character 7), head and neck region (characters 8–16), trunk (characters 17–23), foot, pedal glands and toes (characters 24–37), sensory system (characters 38–50) and trophi (characters 51–60). The respective character states for each species are presented in Tables 3–8.

### Identification characters

#### Habitat and behaviour (Table 3)

1. Habitat

a. freshwater (exemplar *S. grandis*)

b. brackish (exemplar *S. bicornis*)

c. marine (exemplar *S. atlantica*)

d. inland saline (exemplar *S. cylindrica*)

2. Swimming duration

a. exclusively pelagic (exemplar *S. pectinata*) or only adheres to objects transiently when disturbed (exemplar *S. oblonga*)

b. interrupted by frequent, long-lasting adherences to diverse objects (e.g., plants; exemplar *S. tremula*)

3. Adherence to diverse objects

a. absent (exemplar *S. pectinata*) or only transiently and only when disturbed (exemplar *S. oblonga*)

b. long-lasting adherence without any twisting movement about the longitudinal axis (exemplar *S. kitina*)

c. long-lasting adherence combined with a twisting movement about the longitudinal axis (exemplar *S. tremula*)

**Figure 3.**
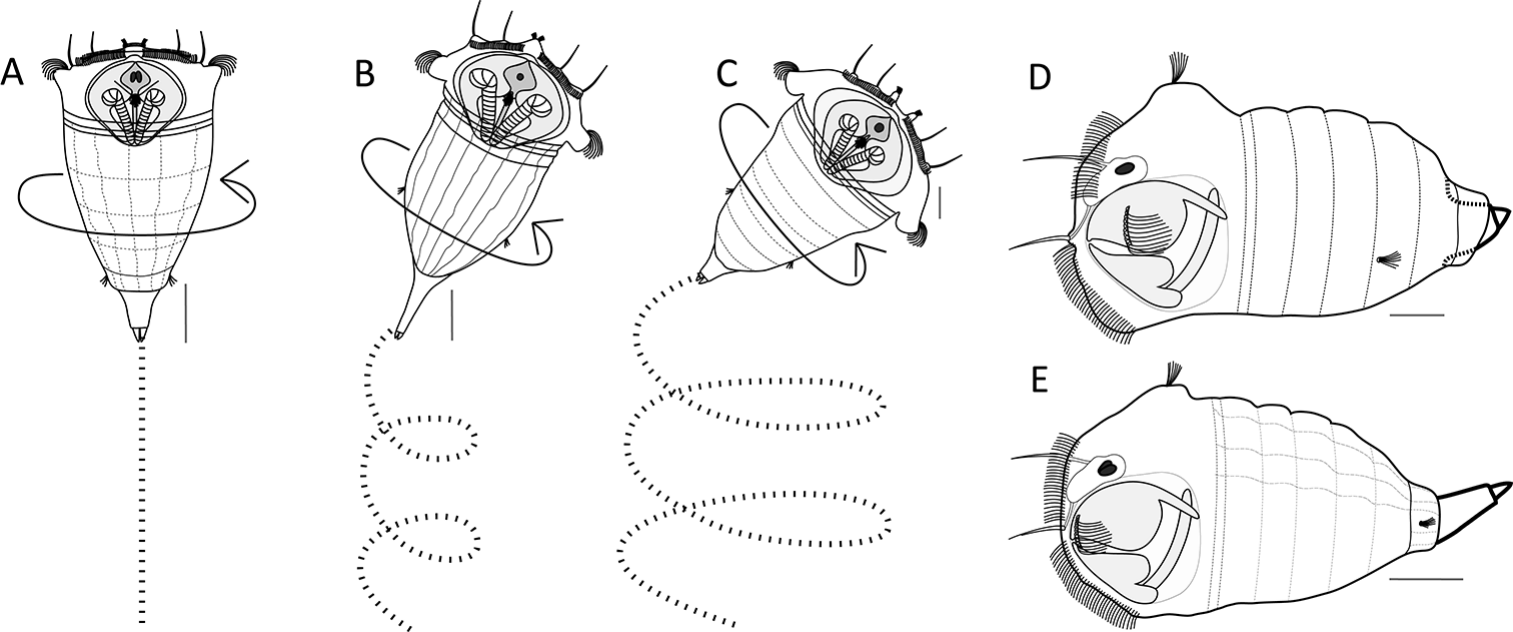
Habitat and behaviour. **A–C** Examples of the swimming behaviour **A** straight (*S. tremula*) **B** slightly coiled (*S. stylata*) **C** distinctly coiled (*S. pectinata*) **D–E** Foot position (thick lines) while swimming **D** foot retracted (*S. baltica*) **E** foot not retracted (*S. tremula*). Scale bar: 50 µm.

4. Swimming motion (always combined with a rotation about the longitudinal axis)

a. in a straight line (Fig. 3A; exemplar *S. tremula*, *S. kitina*)

b. slightly coiled (Fig. 3B; exemplar *S. stylata*, *S. tremuloida*)

c. distinctly coiled (Fig. 3C; exemplar *S. pectinata*, *S. grandis*)

5. Foot position while swimming

a. partly or fully retracted (Fig. 3D; exemplar *S. baltica*)

b. not retracted (Fig. 3E; exemplar *S. tremula*)

6. Directional changes while swimming

a. many sudden directional changes (exemplar *S. stylata, S. oblonga*)

b. few or no sudden directional changes (exemplar *S. pectinata*)

#### Size (Table 3)

7. Overall body length of mature specimens (measured from the apical field to the distal tips of the toes, excluding the cilia)

a. less than 250 µm

b. more than 250 µm

#### Head and neck region (Table 4)

**Figure 4.**
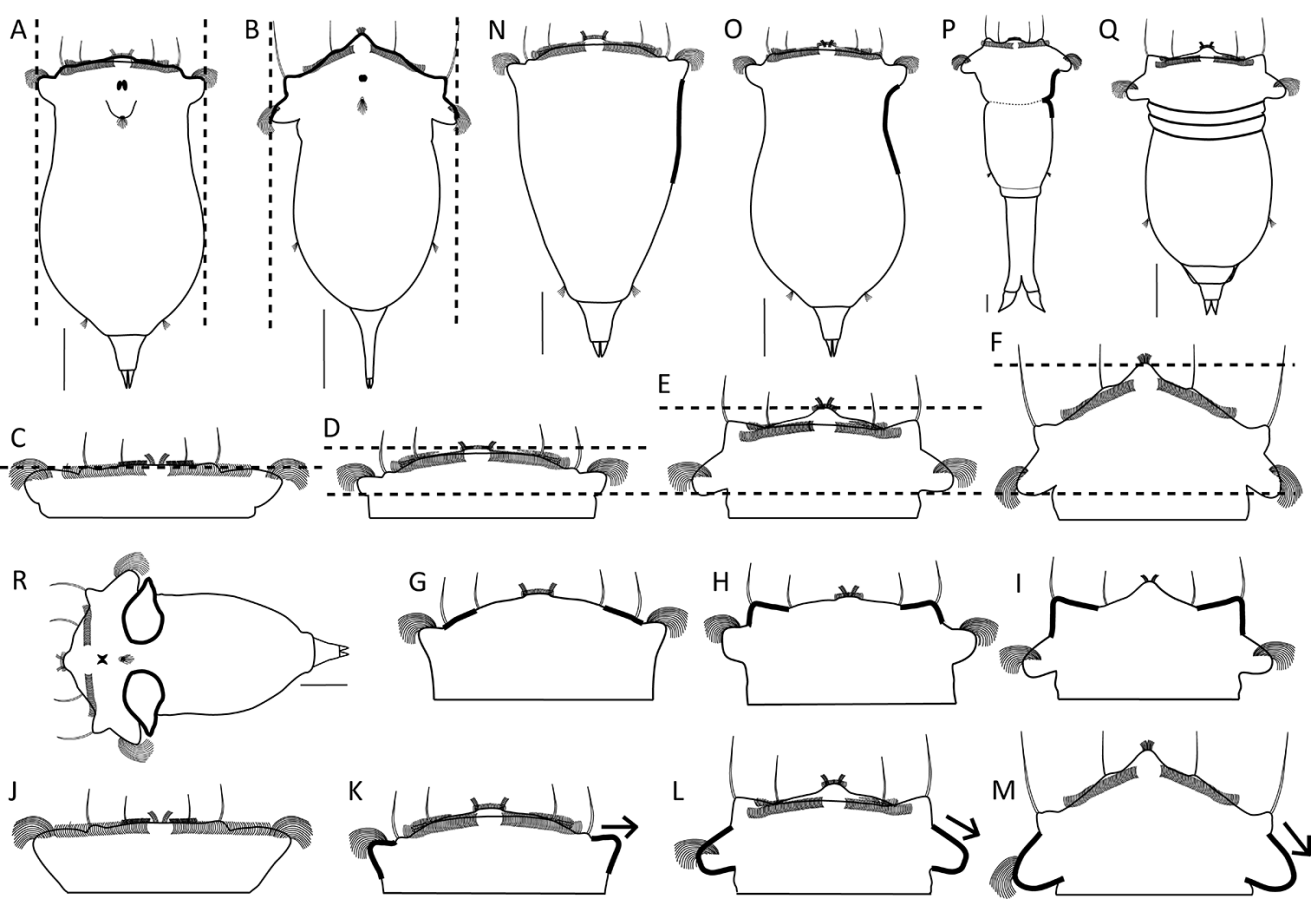
Head and neck region. **A, B** Relative width of the apical field (dashed lines) **A** as wide as the trunk (*S. tremuloida*) **B** wider than the trunk (*S. longipes*) **C–F** Elevation of the apical field (top line) relative to the auricles (bottom line) **C** level (*S. grimpei*) **D** slightly elevated (*S. tremula*) **E** intermediate (*S. oblonga*) **F** strongly elevated (*S. grandis*) **G–I** Elevation of the dorsolateral styles (thick lines) **G** not or very slightly raised (*S. tremula*) **H** intermediate (*S. gyrina*) **I** strongly raised (*S. baltica*) **J–M** Auricle size (thick lines) and orientation (arrows) **J** not clearly demarcated from rotatory organ, directed laterally (*S. grimpei*) **K** small, directed laterally (*S. tremula*) **L** medium, directed semi-caudally (*S. oblonga*) **M** large, directed caudally (*S. grandis*) **N–Q** Separation of the head and trunk region **N** gradual transition, the head is not distinctly offset from the trunk (*S. tremula*) **O** head and trunk are demarcated by the narrower neck (*S. tremuloida*) **P** demarcation by a sharp constriction in the neck region (*S. pachypoda*) **Q** by distinct transversal folds (*S. oblonga*) **R** Presence of saccate appendages (thick lines) caudal to auricles (*S. bicornis*). Drawings modified from: **P**Kutikova and Vassiljeva (1982)**R**Koste (1981). Scale bars: 50 µm.

8. Apical field – Width in relation to the trunk width^1^

a. as wide as the trunk (Fig. 4A; exemplar *S. tremuloida*)

b. wider than the trunk (Fig. 4B; exemplar *S. longipes*, *S. triophthalma*)

9. Apical field – Elevation with respect to auricles

a. level (Fig. 4C; exemplar *S. grimpei*) to slightly elevated (Fig. 4D; exemplar *S. tremula*)

b. intermediate (Fig. 4E; exemplar *S. triophthalma*)

c. strongly elevated; distinctly convex (Fig. 4F; exemplar *S. grandis*)

10. Dorsolateral styles – Elevation

a. not raised to very slightly raised (Fig. 4G; exemplar *S. tremula*)

b. intermediate (Fig. 4H; exemplar *S. gyrina*)

c. strongly raised (Fig. 4I; exemplar *S. baltica*)

11. Auricles – Size

a. not clearly distinct from the rotatory organ (Fig. 4J; exemplar *S. grimpei*)

b. small (Fig. 4K; exemplar *S. tremula*)

c. medium (Fig. 4L; exemplar *S. oblonga*)

d. large (Fig. 4M; exemplar *S. grandis*)

12. Auricles – Orientation

a. directed laterally (Fig. 4J, K; exemplar *S. tremula*)

b. directed semi-caudally (Fig. 4L; exemplar *S. oblonga*)

c. directed caudally (Fig. 4M; exemplar *S. grandis*)

13. Neck region – Demarcation of the head and trunk regions

a. gradual transition from the head into the trunk region; the neck is neither constricted nor distinctly offset (Fig. 4N; exemplar *S. tremula*)

b. demarcated; the neck is narrower than the head and trunk (Fig. 4O; exemplar *S. tremuloida*)

c. demarcated by a sharp constriction (Fig. 4P; exemplar *S. pachypoda*) or by distinct transversal folds (Fig. 4Q; exemplar *S. oblonga*)

14. Saccular appendages at the neck region (that compensate for pressure changes in the body fluid through contraction of the body)

a. absent (Fig. 4A, B; exemplar *S. pectinata*)

b. present (Fig. 4R; exemplar *S. arcifera*, *S. bacillifera*, *S. bicornis*, *S. fennica*)

15. Saccular appendages – Location

a. ventral (exemplar *S. bacillifera*)

b. dorsal (exemplar *S. arcifera*, *S. bicornis*, *S. fennica*)

c. absent (exemplar *S. pectinata*)

**Figure 5.**
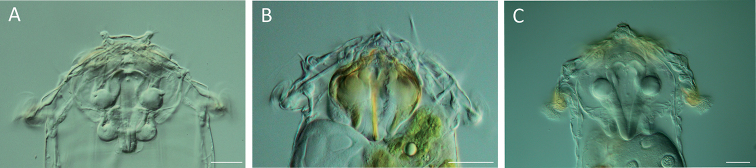
Head and neck region. **A–C**LM images showing different colours in the head region **A** colourless / transparent (*S. pectinata*) **B** mastax moderately yellow or orange (*S. longipes*) **C** parts of rotatory organ or auricles slightly yellow to orange (*S. grandis*). Scale bar: 25 µm.

16. Head region – Colour^2^

a. colourless / transparent (Fig. 5A; exemplar *S. pectinata*)

b. mastax or parts thereof moderately yellow or orange (Fig. 5B; exemplar *S. longipes*)

c. parts of rotatory organ or auricles slightly yellow to orange (Fig. 5C; exemplar *S. grandis*)

#### Trunk (Table 5)

**Figure 6.**
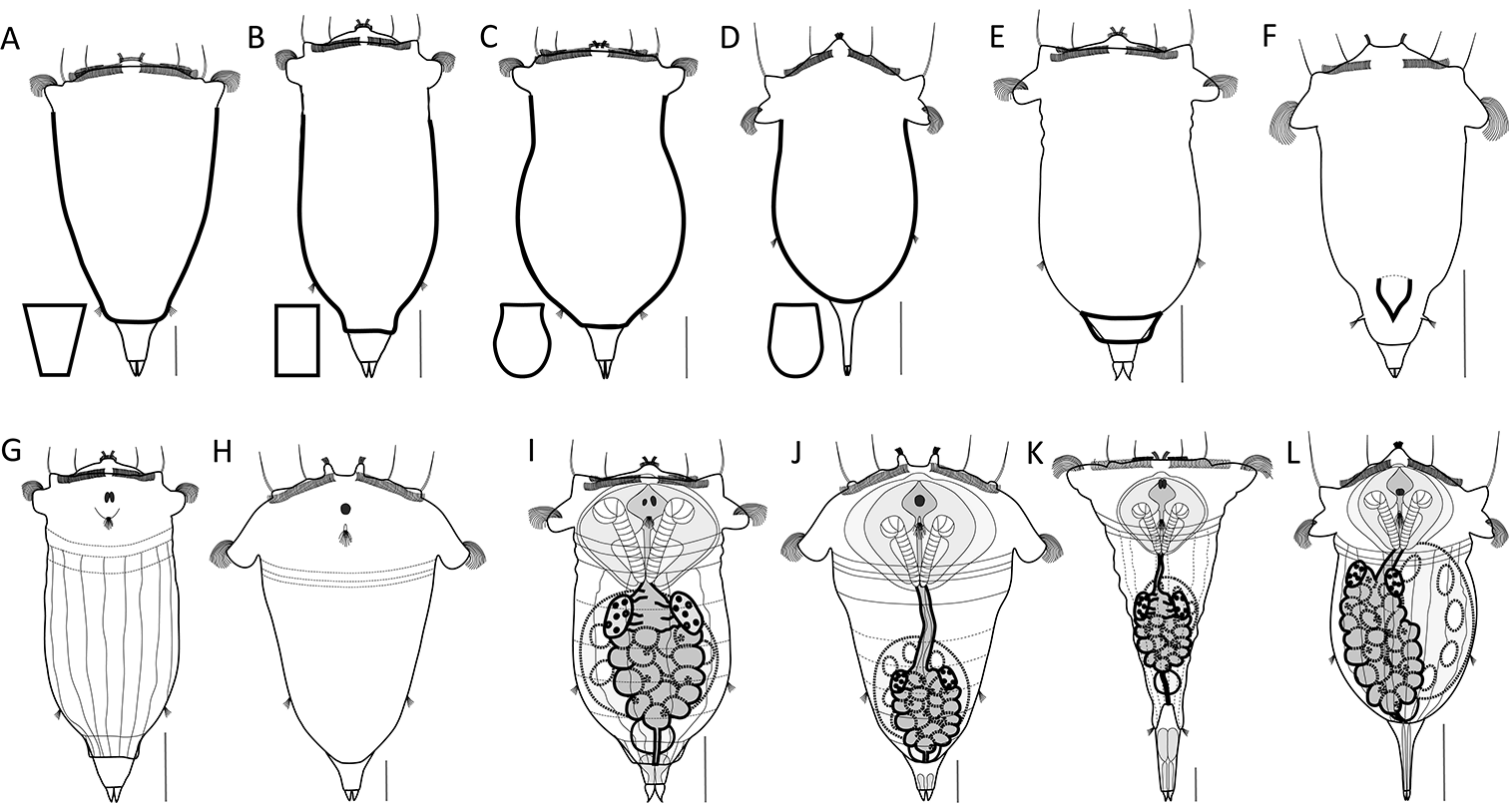
Trunk region. **A–D** Morphology of the trunk region (thick lines) **A** conical (*S. tremula*) **B** cylindrical (*S. tavina*) **C** bell-shaped (*S. tremuloida*) **D** wineglass-shaped (*S. longipes*) **E** Presence of a distinct anal pseudosegment (thick line; *S. oblonga*) **F** Presence of a posteriodorsal saccate appendage (thick line; *S. arcifera*) **G, H** Longitudinal striae on the dorsal trunk **G** present (*S. tavina*) **H** absent (*S. pectinata*) **I–L** Location of the internal organs **I** occupy entire trunk region (*S. oblonga*) **J** occupy middle and caudal trunk regions, oesophagus only structure in the anterior trunk (*S. pectinata*) **K** occupy middle trunk region, cloaca ends anteriorly to the lateral antennae (*S. grimpei*) **L** stomach and ovary each occupy separate sides of the trunk (*S. longipes*). Drawings modified from **B, G**Rousselet (1902)**F**Rougier and Pourriot (2006). Scale bar: 50 µm.

17. Trunk region – Shape^3^

a. conical: trunk decreases gradually in width caudally (Fig. 6A; exemplar *S. tremula*)

b. cylindrical: trunk elongate, decreases in width only in its caudal quarter (Fig. 6B; exemplar *S. tavina*)

c. bell- (Fig. 6C: exemplar *S. tremuloida*) to wineglass-shaped (Fig. 6D; exemplar *S. longipes*), trunk is slightly bulbous and narrows abruptly in its caudal third.

18. Anal pseudosegment

a. distinct anal pseudosegment present (Fig. 6E; exemplar *S. oblonga*)

b. anal pseudosegment barely visible or absent (Fig. 6A–D; exemplar *S. longipes*)

19. Posteriodorsal saccate appendage on the integument (that compensates for pressure changes in the body fluid through contraction of the body)

a. present (Fig. 6F; exemplar *S. arcifera*)

b. absent (Fig. 6A–E; exemplar *S. longipes*)

20. Longitudinal striae on the dorsal trunk

a. present (Fig. 6G; exemplar *S. tavina*)

b. absent (Fig. 6H; exemplar *S. pectinata*)

**Figure 7.**
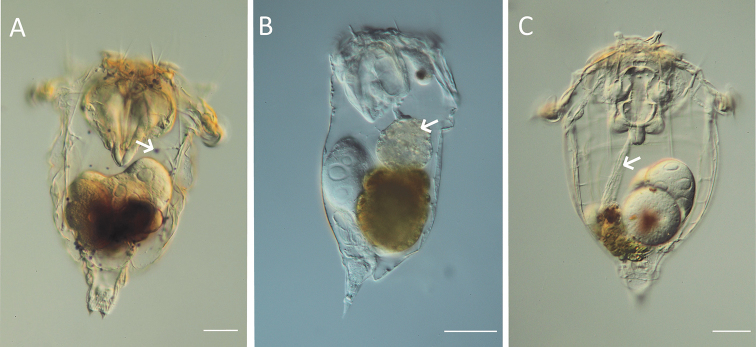
LM images of species of *Synchaeta*. **A** Presence of violet globules in the body cavity (arrow; *S. baltica*) **B, C**LM images of the habitus showing different morphologies of the oesophagus (arrows) **B** oesophagus widens to form a proventriculus (*S. tremuloida*) **C** oesophagus highly tensile with numerous longitudinal striae (*S. pectinata*). Scale bar: 50 µm.

21. Internal organs – Location

a. occupy entire trunk region (Fig. 6I; exemplar *S. oblonga*)

b. occupy middle and caudal trunk regions; the oesophagus is the only structure present in the anterior trunk region (Figs 6J, 7C; exemplar *S. pectinata*)

c. occupy middle trunk region; cloaca ends in posterior quarter of trunk, anteriorly to the lateral antennae (Fig. 6K; exemplar *S. grimpei*)

d. stomach and ovary each occupy separate sides of the trunk (Fig. 6L; exemplar *S. longipes*)

22. Violet globules in the body cavity

a. present (Fig. 7A, arrow; only known for *S. baltica*, *S. bicornis*, and *S. grimpei*, where the globules can also be absent)

b. absent (Fig. 7B, C; exemplar *S. pectinata*)

23. Oesophagus – Morphology

a. short oesophagus that widens in its caudal half to form a proventriculus (Fig. 7B; exemplar *S. tremula*, *S. tremuloida*)

b. highly tensile oesophagus, narrow or broad and of equal width, with numerous longitudinal striae (Fig. 7C; exemplar *S. pectinata*, *S. baltica*)

#### Foot, pedal glands and toes (Table 6)

**Figure 8.**
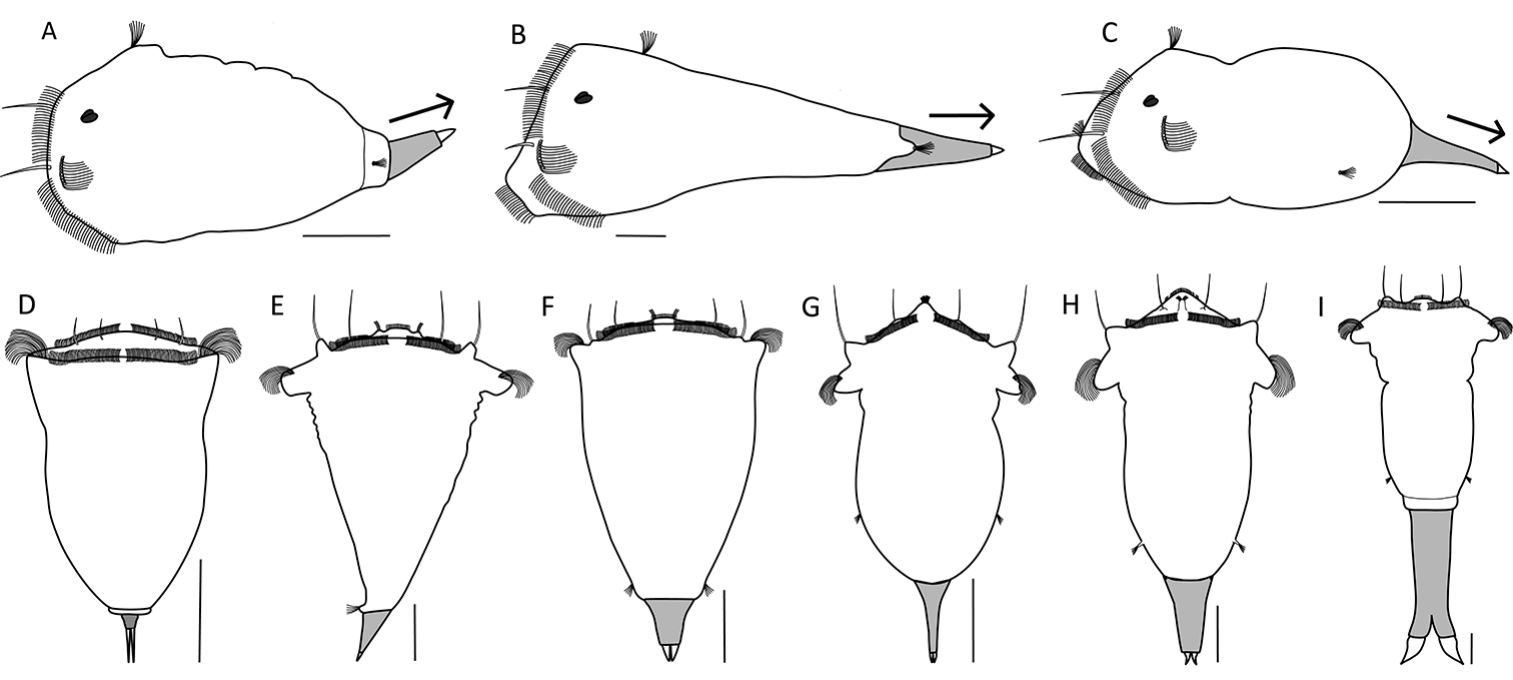
Foot shape. **A–C** Orientation of the foot (grey areas and arrows) **A** directed dorsally (*S. tremula*) **B** coplanar with the longitudinal axis (*S. grimpei*) **C** directed ventrally (*S. longipes*) **D–I** Shape and size of the foot (grey areas) **D** minute, shorter than the toes (*S. atlantica*) **E** triangular, medium (*S. triophthalma*) **F** conical, medium (*S. tremula*) **G** slender, long (*S. longipes*) **H** broad, long (*S. johanseni*) **I** massive, cylindrical (*S. pachypoda*). Drawings modified from: **D**Zelinka (1907)**H**Harring (1921)**I**Kutikova and Vassiljeva (1982). Scale bar: 50 µm.

24. Foot – Orientation

a. directed dorsally (Fig. 8A; exemplar *S. tremula*, *S. baltica*, *S. cecilia*)

b. coplanar with the longitudinal axis of the body or directed very slightly ventrally (Fig. 8B; exemplar *S. grimpei*)

c. directed ventrally (Fig. 8C; exemplar *S. longipes*)

25. Foot – Shape

a. minute, less than one-tenth of the overall body length; as long as or shorter than the toes (Fig. 8D; exemplar *S. atlantica*)

b. triangular, medium; between one-fourth and one-sixth of the overall body length (Fig. 8E; exemplar *S. triophthalma*)

c. conical, short to medium; less than one-fourth of the overall body length, but always longer than the toes (Fig. 8F; exemplar *S. tremula*)

d. slender, medium to long; ca. one-third to one-fifth of the overall body length (Fig. 8G; exemplar *S. longipes*)

e. broad, long; ca. one-third of the overall body length (Fig. 8H; exemplar *S. johanseni*, *S. baltica*)

f. cylindrical, massive; approximately one-half of the overall body length (Fig. 8I; exemplar *S. pachypoda*, *S. pachypoida*

**Figure 9.**
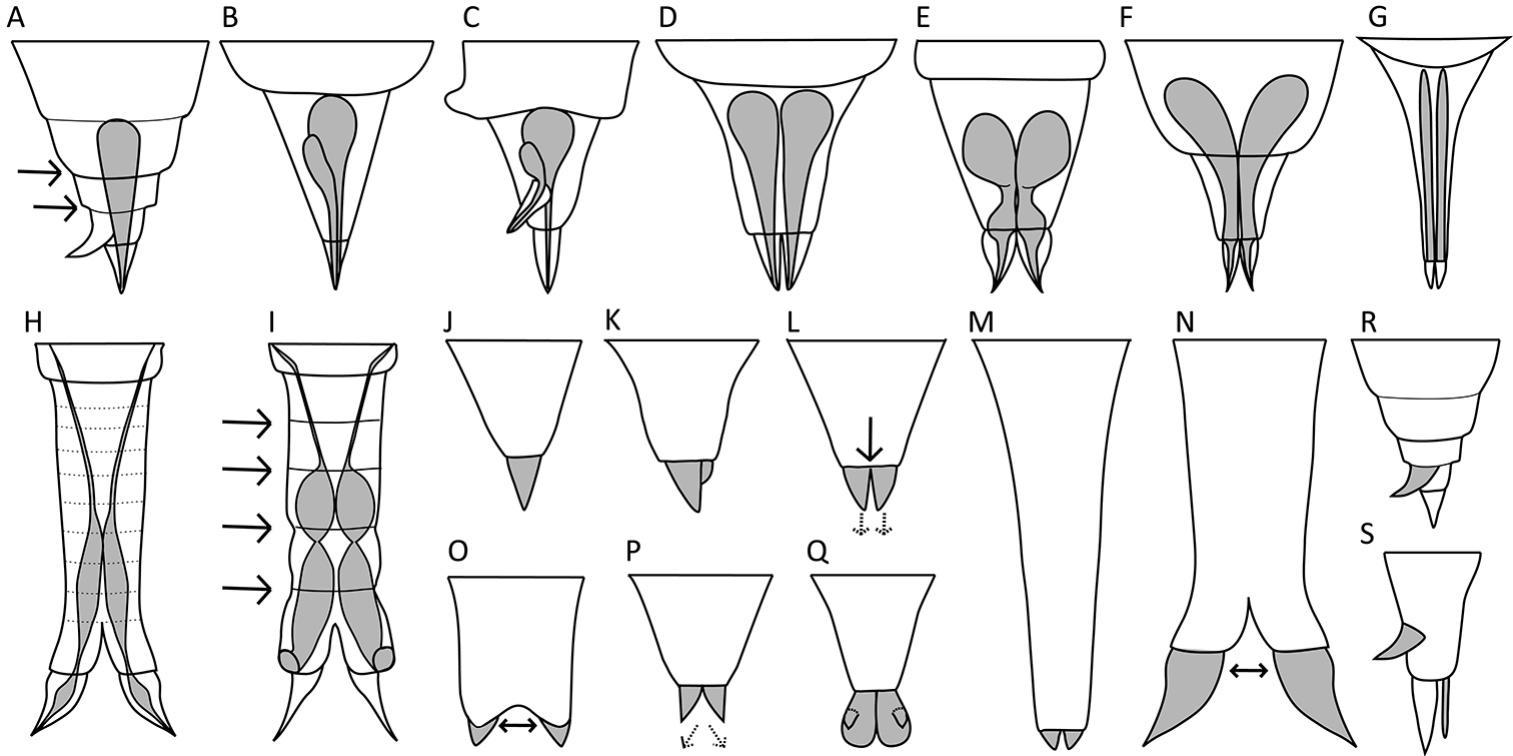
Foot, pedal glands and toes. **A–I** Presence of pseudosegments (arrows) and morphology of pedal glands (grey areas) **A** foot pseudosegmented, pedal gland single and of foot-length (*S. neapolitana*) **B** glands asymmetrical with the larger one being of foot-length, glands terminating in the toes (*S. triophthalma*) **C** glands asymmetrical with one gland terminating in the toe and one in the dorsal spur (*S. hutchingsi*) **D** symmetrical glands of foot-length, voluminous proximally and decreasing gradually in width moving caudally (*S. tremula*) **E** glands shorter than the foot, each spherical proximally and abruptly decreasing caudally before widening again to form a reservoir (*S. oblonga*) **F** glands longer than the foot (*S. prominula*), **G** glands of foot-length and tubular (*S. longipes*) **H** foot with wrinkles, glands tubular, suspended from the trunk (*S. pachypoda*) **I** foot pseudosegmented, each gland with two voluminous sections, suspended from the trunk, glands terminate proximally to the toes (*S. pachypoida*) **J–Q** Symmetry, size and separation of the toes **J** single toe (*S. triophthalma*) **K** asymmetrical, toes of different shape (*S. cecilia*) **L** symmetrical toes of medium size, bases of the toes are in contact (arrow), tips are close to one another or very slightly divergent (dashed arrows; *S. tremula*) **M** toes symmetrical, minute to small (*S. grandis*) **N** toes symmetrical, very large (*S. pachypoda*) **N, O** Bases of the toes widely separated (arrow; *S. pachypoda, S. baltica*) **P** bases of the toes in contact, tips distinctly divergent (dashed arrows; *S. gyrina*) **Q** toes without tips, squamate (*S. squamadigitata*) **R, S** Additional appendages of the foot (grey areas) **R** dorsolateral spur (dorsal view; *S. neapolitana*) **S** ventral spine and dorso-lateral spur (lateral view; *S. hutchingsi*). Drawings modified from: **A, R**Lie-Pettersen (1905)**K**Rousselet (1902)**F, H–I, N**Kutikova and Vassiljeva (1982)**Q**De Smet (2006).

26. Pedal glands – Symmetry

a. asymmetrical; either of different size and shape or only singly present (Fig. 9A–C; exemplar *S. cecilia, S. triophthalma*)

b. symmetrical (Fig. 9D–I; exemplar *S. tremula*, *S. pectinata*)

27. Pedal gland(s) – Number and arrangement^4^

a. one single pedal gland (Fig. 9A; exemplar *S. neapolitana*)

b. pedal glands are paired but of different size and shape; one is rudimental (Fig. 9B–C; exemplar *S. cecilia*, *S. hutchingsi*, *S. tamara*, *S. triophthalma*)

c. two symmetrical glands are present (Fig. 9D–I; exemplar *S. tremula*, *S. pectinata*)

28. Pedal gland(s) – Length^5^

a. shorter than the foot (Fig. 9E; exemplar *S. oblonga*)

b. as long as the foot (Fig. 9A–D; exemplar *S. tremula*)

c. longer than the foot, extending into the caudal trunk region (Fig. 9F; exemplar *S. atlantica*, *S. prominula*, *S. rousseleti*)

29. Pedal gland(s) – Shape

a. tubular; of even width along their entire length (Fig. 9G; exemplar *S. longipes*)

b. club-shaped; voluminous proximally, decreasing gradually caudally (Fig. 9A, D; exemplar *S. tremula*)

c. voluminous proximally, decreasing abruptly caudally before widening again to form a reservoir in the distal half (Fig. 9E; exemplar *S. oblonga*)

d. tubular, suspended from the trunk (Fig. 9H; exemplar *S. pachypoda*)

e. each gland possesses two large and voluminous sections that are demarcated by a narrowing from one another; suspended from the trunk proximally (Fig. 9I; exemplar *S. grimpei*, *S. pachypoida*)

30. Pedal gland(s) – Opening

a. into the tip(s) of the toe(s) (Fig. 9A, B, D–H; exemplar *S. tremula*, *S. pectinata*)

b into a toe with the second into a spur (Fig. 9C; exemplar *S. hutchingsi*)

c. at the base of the toes (Fig. 9I; exemplar *S. pachypoida*)

31. Toes – Symmetry

a. asymmetrical; only one toe is present (Fig. 9J; exemplar *S. hutchingsi*, *S. neapolitana*, *S. triophthalma*) or two toes are of different size and shape (Fig. 9K; exemplar *S. cecilia*)

b. symmetrical paired toes (Fig. 9L–Q; exemplar *S. tremula, S. pectinata*)

32. Toe(s) – Number and arrangement

a. only one toe is present (Fig. 9J; exemplar *S. hutchingsi*, *S. neapolitana*, *S. triophthalma*)

b. paired toes present that are of different size and shape; one can be rudimental (Fig. 9K; exemplar *S. cecilia*, *S. tamara*)^6^

c. paired toes of equal size and shape (Fig. 9L–Q; exemplar *S. tremula*, *S. pectinata*)

33. Toe(s) – Size in relation to foot length

a. minute to small; less than one-tenth of the overall foot length (Fig. 9M; exemplar *S. grandis*)

b. medium to large; between one-tenth to one-quarter of the foot length (Fig. 9J–L; exemplar *S. tremula*)

c. very large; at least one-third of the foot length (Fig. 9N; exemplar *S. pachypoda*)

34. Toe(s) – Proximal separation

a. bases of the toes are widely separated (Fig. 9N, O; exemplar *S. baltica, S. pachypoda*)

b. bases of the toes are close to or in contact with one another (Fig. 9K–M, P, Q; exemplar *S. tremula*, *S. pectinata*)

c. only one toe is present (Fig. 9J; exemplar *S. hutchingsi*, *S. neapolitana*, *S. triophthalma*)

35. Toe(s) – Distal separation

a. tips are close to one another or only very slightly divergent (Fig. 9L; exemplar *S. tremula*)

b. tips are widely separated, distinctly divergent (Fig. 9P; exemplar *S. gyrina*)

c. only one toe is present (Fig. 9J; exemplar *S. hutchingsi*, *S. neapolitana*, *S. triophthalma*)

d. toes without tips; squamate, with rounded distal margin (Fig. 9Q; exemplar *S. squamadigitata*)

36. Additional foot appendages

a. none (Fig. 9J–Q; exemplar *S. tremula, S. pectinata*)

b. dorsolateral spur present (Fig. 9R; exemplar *S. neapolitana*)

c. ventral spine and dorsolateral spur present (Fig. 9S; exemplar *S. hutchingsi*)

37. Foot – Number of “pseudosegments”^7^

a. at least two (Fig. 9A, I; exemplar *S. neapolitana*, *S. pachypoida*, *S. squamadigitata*, *S. tamara*)

b. only one (Fig. 9B–H; exemplar *S. tremula*)

#### Sensory system (Table 7)

**Figure 10.**
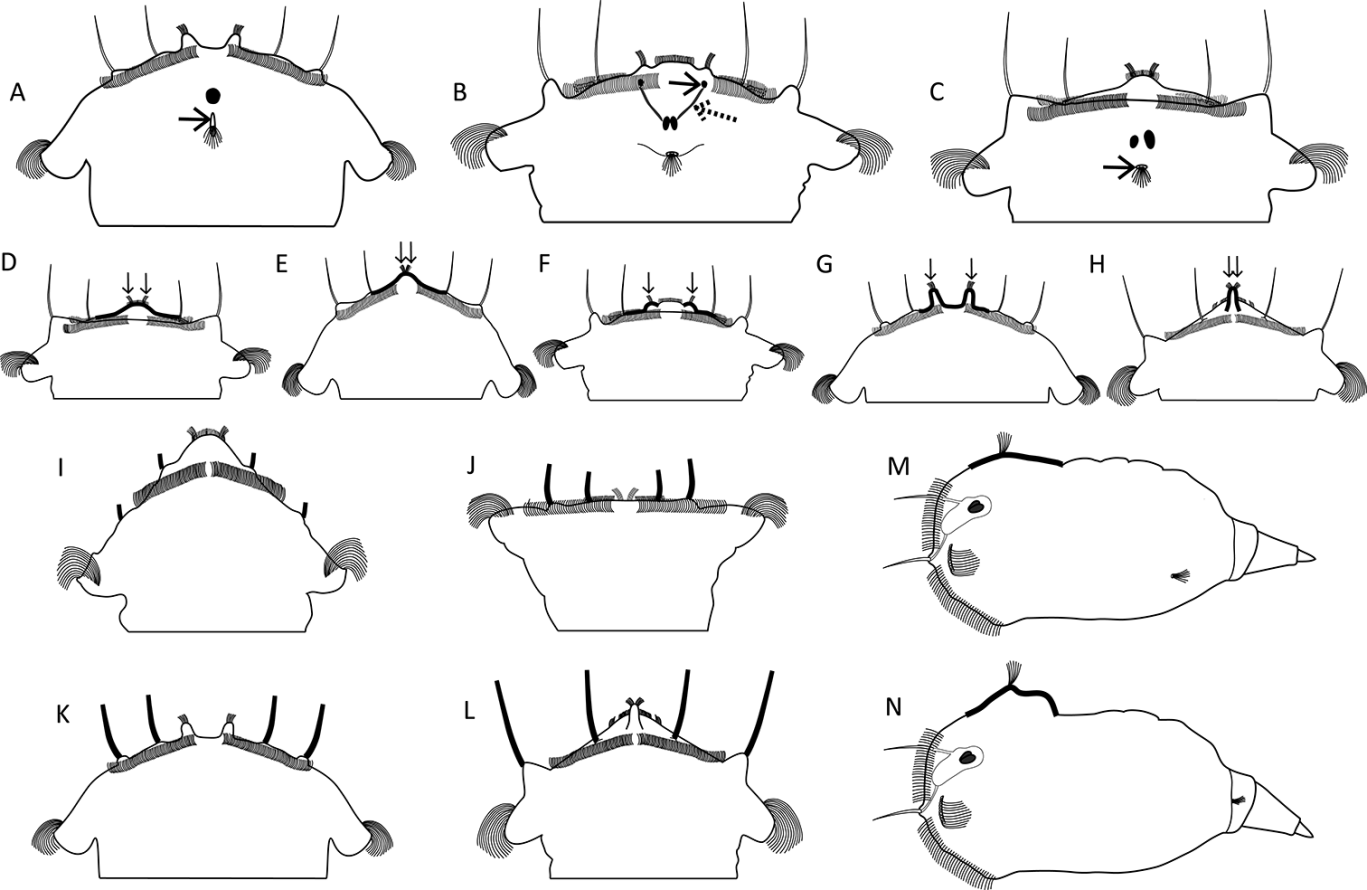
Sensory system. **A–C** Head region showing the cerebral eye, pigment granules and the opening of the dorsal antenna **A** cerebral eye single, dorsal antenna opening slit-shaped (arrow; *S. pectinata*) **B** two partially fused cerebral eyes, frontal aggregations (arrow) and streams (dashed arrow) of pigment granules present (*S. triophthalma*) **C** cerebral eyes distinctly separated, dorsal antenna opening round (arrow; *S. oblonga*) **D–H** Morphology of the apical receptors (thickened lines, arrows) **D** receptors slightly separated, situated on a slight elevation centrally on the apical field (*S. oblonga*) **E** receptors incompletely separated, situated on a strong elevation centrally on the apical field (*S. grandis*) **F** receptors distinctly separated, each situated on a bulge (*S. triophthalma*) **G** receptors distinctly separated, each situated on a strong tentacle-like elevation (*S. pectinata*) **H** receptors incompletely separated, situated on a single tubular elevation (*S. vorax*) **I–L** Lengths of the lateral and dorsolateral styles (thickened lines) **I** minute (*S. squamadigitata*) **J** short (*S. grimpei*) **K** medium (*S. pectinata*) **L** long (*S. vorax*) **M, N** Elevation underlying the dorsal antenna (thickened lines) **M** not elevated to slightly elevated (*S. oblonga*) **N** distinct prominence (*S. tremuloida*). Drawings modified from: **I**De Smet (2006).

38. Cerebral eye – Morphology

a. single (Fig. 10A; exemplar *S. pectinata*)

b. paired but partially fused or connected by pigment granules (Fig. 10B; exemplar *S. triophthalma*)

c. paired and distinctly separated from one another (Fig. 10C; exemplar *S. oblonga*, *S. lakowitziana*)

**Figure 11.**
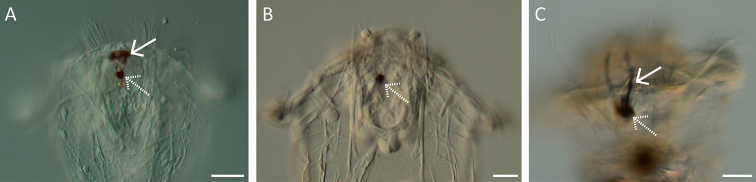
Cerebral eye and pigment granules. **A–C**LM images with regard to the cerebral eye (dashed arrows), frontal aggregations and streams of pigment granules (normal arrows) **A** Distinct frontal aggregations of pigment granules present, cerebral eye of normal size (*S. triophthalma*) **B** Frontal aggregations and streams of pigment granules are absent, cerebral eye of normal size (*S. pectinata*) **C** distinct streams of pigment granules are present, large cerebral eye (*S. baltica*). Scale bar: 20 µm.

39. Cerebral eye – Size

a. small to medium, evenly shaped (Fig. 11A, B; exemplar *S. pectinata*)

b. large, irregularly shaped (Fig. 11C; exemplar *S. baltica*, *S. hutchingsi*)

40. Frontal aggregations of pigment granules^8^

a. present (Figs 10B, 11A; exemplar *S. triophthalma*)

b. absent (Figs 10A, C, 11B; exemplar *S. pectinata*)

41. Streams of pigment granules to the anterior margin of the apical field^9^

a. present (Figs 10B, 11C; exemplar *S. triophthalma*, *S. baltica*)

b. absent (Figs 10A, C, 11B; exemplar *S. pectinata*)

42. Apical receptors – Separation

a. Two ciliary tufts, the bases of which are not completely separated from one another (Fig. 10E, H; exemplar *S. grandis*, *S. vorax*)

b. Two ciliary tufts, the bases of which are slightly separated from one another (Fig. 10D; exemplar *S. oblonga*)

c. Two ciliary tufts that are distinctly separated from one another (Fig. 10F, G; exemplar *S. triophthalma*, *S. pectinata*)

43. Apical receptors – Elevation

a. on a flat or slight central elevation of the apical field (Fig. 10D; exemplar *S. oblonga*)

b. on a strong central elevation of the apical field (Fig. 10E; exemplar *S. grandis*)

c. on two bulges or pimples (Fig. 10F; exemplar *S. triophthalma*)

d. on strong, paired elevations (tentacles) (Fig. 10G; exemplar *S. pectinata*)

e. on a single, tubular elevation (Fig. 10H; exemplar *S. fennica*, *S. johanseni*, *S. vorax*)

44. Lateral and dorsolateral styles – Length^10^

a. minute (Fig. 10I; exemplar *S. squamadigitata*)

b. short (Fig. 10J; exemplar *S. grimpei*)

c. medium (Fig. 10K; exemplar *S. pectinata*)

d. long (Fig. 10L; exemplar *S. vorax*)

45. Dorsal antenna – Elevation

a. none to a slight elevation (Fig. 10M; exemplar *S. oblonga*)

b. distinct prominence to a snout-like projection (Fig. 10N; exemplar *S. tremuloida*)

**Figure 12.**
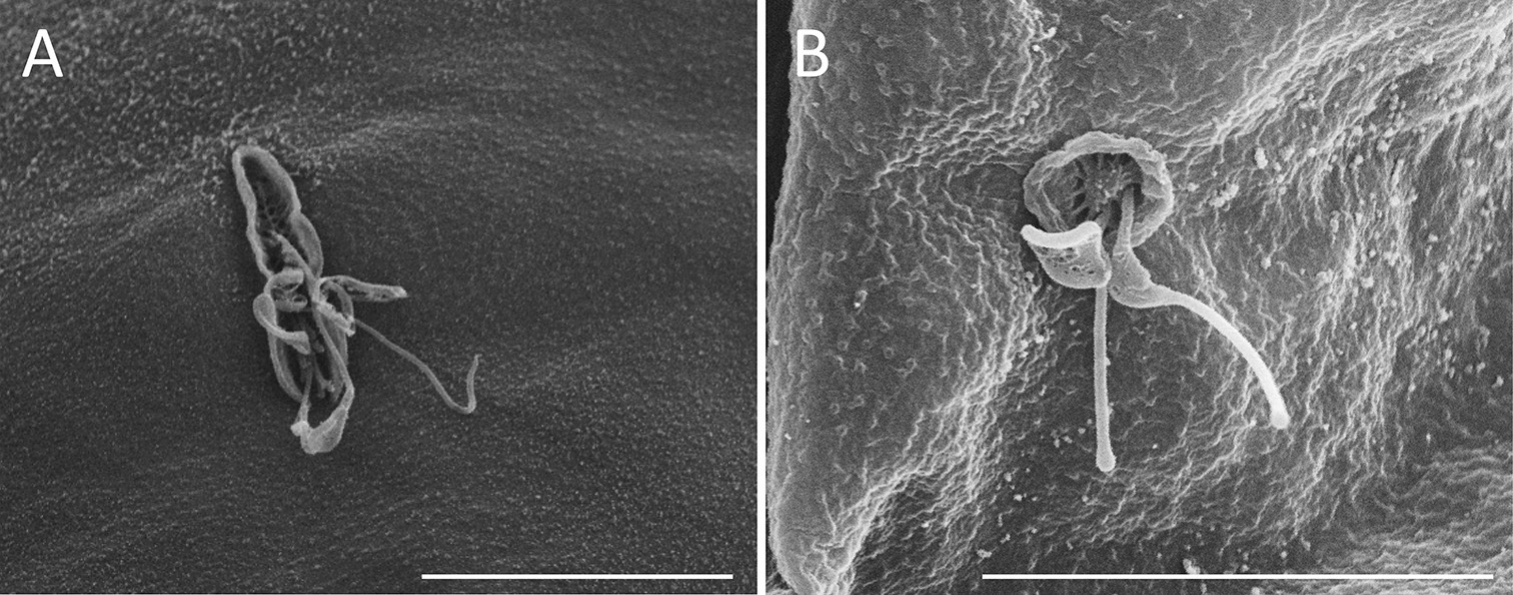
Dorsal antenna. **A, B** REM images of the basal opening of the dorsal antenna **A** slit-shaped (*S. pectinata*) **B** round (*S. tremula*). Scale bar: 10 µm.

46. Dorsal antenna – Basal opening

a. slit-shaped, longer than wide (Figs 10A, 12A; exemplar *S. grandis*, *S. pectinata*)

b. round (Figs 10B–C, 12B; exemplar *S. tremula*)

**Figure 13.**
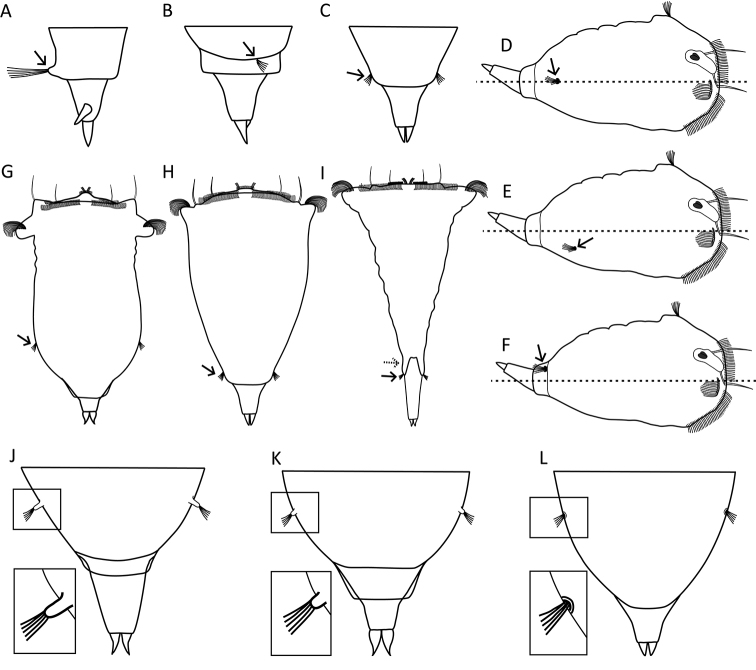
Location and morphology of the lateral antennae. **A–C** Number and size of the lateral antenna(e) (arrow) **A** single, enlarged left lateral antenna (*S. hutchingsi*) **B** single, right lateral antenna (*S. tamara*) **C** lateral antennae paired, symmetrical, and of normal size (*S. tremula*) **D–F** Location of the lateral antenna(e) (arrows) relative to the median transversal axis (dashed line) (lateral habitus is presented as a stylized drawing that is species independent) **D** directly lateral (e.g., *S. tremula*) **E** ventrolateral (e.g., *S. oblonga*) **F** mid-dorsal, single antenna slightly displaced to the right of the body axis (e.g., *S. tamara*) **G–I** Location of the lateral antennae relative to the longitudinal axis (arrows) **G** in the posterior third of the trunk region (*S. oblonga*) **H** in the caudal-most trunk region at or near the base of the foot (*S. tremula*) **I** on lateral lobes (dashed arrow) caudally to the cloaca and in the proximal third of the foot (*S. grimpei*) **J–L** The base of the lateral antennae (detail in inset) **J** surrounded by a tubular epidermal fold (*S. johanseni*) **K** surrounded by a papillary epidermal fold (*S. oblonga*) **L** surrounded by a low epidermal fold (*S. pectinata*). Drawings modified from: **B**Smirnov (1933) and Friedrich and De Smet (2000)**J**Harring (1921).

47. Lateral antenna(e) – Number

a. one; left lateral antenna is enlarged, right one is absent (Fig. 13A; exemplar *S. hutchingsi*, *S. triophthalma*)

b. one; right lateral antenna of normal size, left one is absent (Fig. 13B; exemplar *S. tamara*)

c. paired symmetrical lateral antennae of normal size (Fig. 13C; exemplar *S. tremula*)

48. Lateral antenna(e) – Location relative to the median transversal axis of the body

a. directly lateral on the median transverse axis (Fig. 13D; exemplar *S. tremula*)

b. ventrolateral to the median transverse axis (Fig. 13E; exemplar *S. pectinata*)

c. mid-dorsal and slightly displaced to the right of the body axis (Fig. 13F; exemplar *S. tamara*)

49. Lateral antenna(e) – Location relative to the longitudinal plane

a. in the posterior third of the trunk region (Fig. 13G; exemplar *S. oblonga*)

b. in the caudal-most trunk region at or near the base of the foot (Fig. 13H; exemplar *S. tremula*)

c. on lateral lobes caudally to the cloaca (Fig. 13I; exemplar *S. grimpei*)

50. Lateral antenna(e) – Base

a. surrounded by a tubular (Fig. 13J; exemplar *S. johanseni*) or papillary (Fig. 13K; exemplar *S. oblonga*) epidermal fold

b. surrounded by a low epidermal fold (Fig. 13L; exemplar *S. pectinata*)

#### Trophi (Table 8)

**Figure 14.**
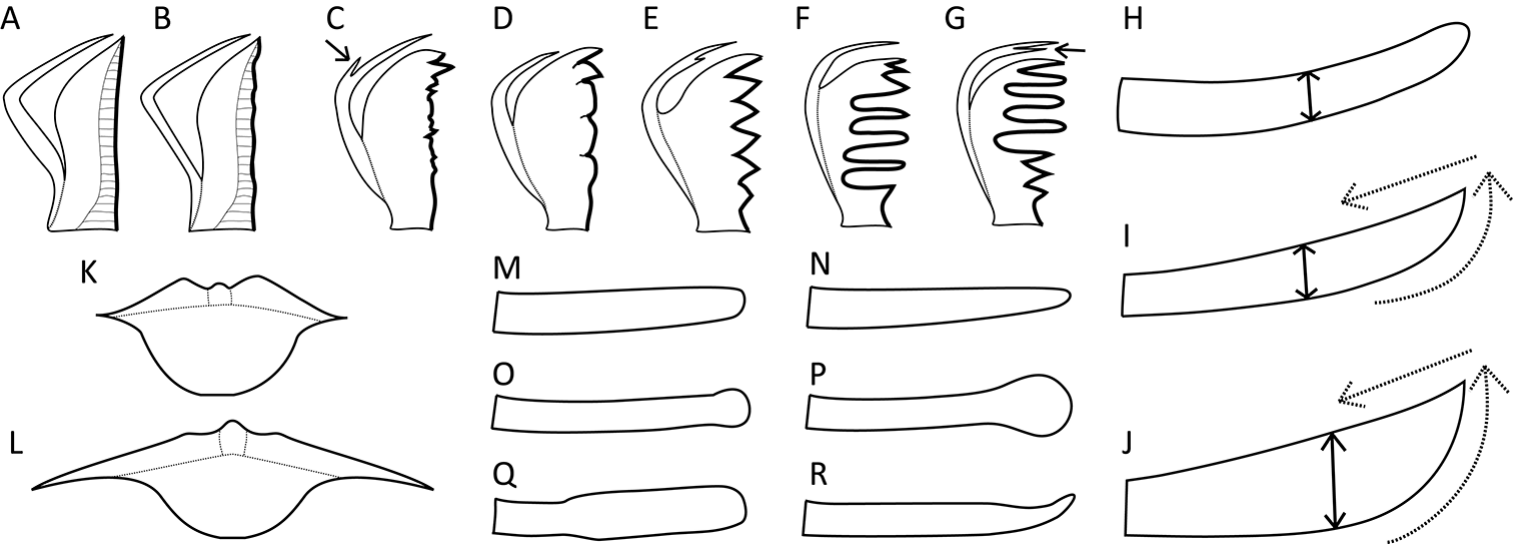
Trophi. **A–G** Morphology of the ramus (“unci”) teeth **A** teeth absent (*S. pectinata*) **B** teeth absent, margin slightly corrugated (*S. stylata*) **C** no distinct teeth, only a serrated plate, frontal hook with a spine (arrow; *S. vorax*) **D** one to two teeth are sharply pointed and remainder are blunt (*S. verrucosa*) **E** teeth distinctly incised, frontal hook with a spine (*S. gyrina*) **F** dorsal teeth distinctly incised and ventral comb-like (*S. triophthalma*) **G** dorsal teeth comb-like and ventral distinctly incised, frontal hook with a spine (arrow; *S. baltica*) **H–J** Shape and breadth (double headed-arrow) of the lateral fulcrum **H** blade-like, narrow, distal end not oblique (*S. tremula*) **I** machete-like, narrow, distal end oblique (dashed arrows; *S. oblonga*) **J** axe-shaped to semi-circular, broad to very broad, distal end oblique (dashed arrows; *S. longipes*) **K–L** Shape of the hypopharynx **K** small to medium, robust (*S. tremula*) **L** broad to very broad, laterally pointed / dagger-like (*S. stylata*) **M–R** Shape of the cauda of the manubrium **M** of even width (*S. baltica*) **N** slightly decreasing distally (*S. pectinata*) **O** with a small knob-like thickening distally (*S. hutchingsi*) **P** with a large knob-like thickening distally (*S. grimpei*) **Q** oar blade shaped at the distal half (*S. oblonga*) **R** cauda spatulate or kinked distally (*S. glacialis*). Drawings modified from: **D**Stemberger (1979) (sub. *S. asymmetrica* Koch-Althaus) and Jersabek et al. (2003a)**R**Friedrich and De Smet (2000).

**Figure 15.**
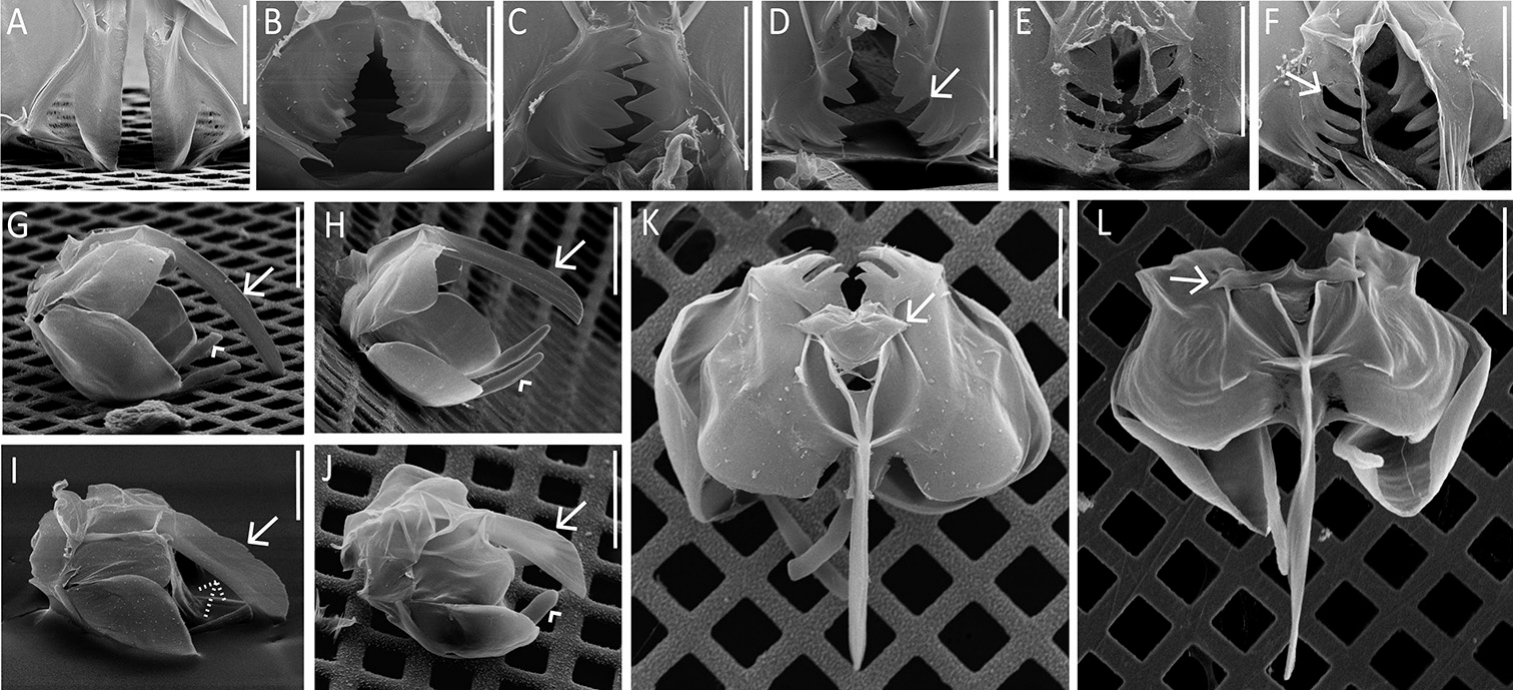
SEM images of the trophi. **A–F** Ramus (“unci”) teeth (ventral sides of the trophi directed upwards) **A** teeth absent (*S. pectinata*) **B** no distinct teeth, only a serrated plate (*S. vorax*) **C** teeth distinctly incised (*S. gyrina*) **D** teeth distinctly incised and separated into two groups by a deep sulcus (arrow; *S. oblonga*) **E** dorsal group of teeth distinctly incised and ventral group of teeth comb-like (*S. triophthalma*) **F** dorsal teeth comb-like and ventral teeth distinctly incised, groups of teeth separated by a deep sulcus (arrow; *S. baltica*) **G–J** Shape of the lateral fulcrum (normal and dashed arrows) and thickness of the cauda (arrow-heads) (trophi from lateral view, ventral sides directed upwards) **G** fulcrum blade-like, narrow, distal end not oblique, cauda of medium thickness (*S. tremula*) **H** fulcrum machete-like, distal end oblique, cauda of medium thickness (*S. oblonga*) **I** fulcrum broad, with distinct dorsal thickening (dashed arrow) and ventral lamella (arrow; *S. vorax*) **J** fulcrum axe-shaped, very broad, with oblique distal end, cauda robust (*S. longipes*) **K, L** Shape of the hypopharynx (arrow) **K** small to medium, robust (*S. tremula*) **L** broad to very broad, laterally pointed / dagger-like (*S. stylata*). Scale bar: 20 µm.

51. Ramus^11^ (“unci”) teeth

a. Edentulous (Figs 14A, 15A; exemplar *S. pectinata*); plate plain, slightly serrated, fringed or corrugated (Fig. 14B, C; exemplar *S. stylata*)

b. With several distinct teeth (Figs 14D–G, 15B–F; exemplar *S. gyrina*, *S. triophthalma*)

52. Ramus*^12^ (“unci”) teeth – Shape

a. teeth absent (Figs 14A, B, 15A; exemplar *S. pectinata*, *S. stylata*)

b. one more or less distinct tooth, remainder serrated (Figs 14C, 15B; exemplar *S. vorax*)

c. one distinctly pointed single tooth, remainder slightly incised and blunt (Fig. 14D; exemplar *S. verrucosa*)

d. all teeth are distinctly incised (Figs 14E, 15C, D; exemplar *S. gyrina, S. oblonga*)

e. dorsal teeth are distinctly incised, ventral teeth are comb-like (Figs 14F, 15E; exemplar *S. triophthalma*)

f. dorsal teeth are comb-like, ventral teeth are distinctly incised (Figs 14G, 15F; exemplar *S. baltica*)

53. Ramus^13^ (“unci”) teeth – Separation

a. teeth are separated into two groups, either by a cleft (Fig. 15D, F, arrow; exemplar *S. oblonga*) and/or by their morphological distinctiveness (Figs 14F, G, 15E, F; exemplar *S. triophthalma*, *S. baltica*)

b. teeth are not separated so that a continuous row of teeth is present (Figs 14E and 15C; exemplar *S. gyrina*)

c. no distinct teeth present (Figs 14A, B, 15A; exemplar *S. pectinata*)

54. Spine of frontal hook^14^

a. absent (e.g., Fig. 14A, B; exemplar *S. pectinata*)

b. present (Fig. 14C, G, arrow; exemplar *S. vorax, S. baltica*)

**Figure 16.**
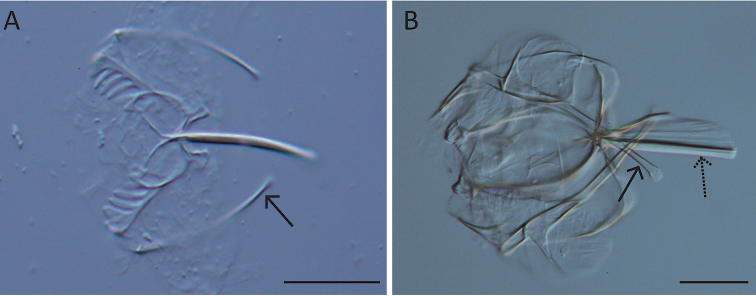
LM images of the trophi. **A, B** Cauda (arrows) and fulcrum (dashed arrow) **A** very thin and slender cauda (*S. kitina*) **B** cauda medium with a large distal knob, fulcrum with dorsal thickening and ventral lamella (*S. grimpei*). Scale bar: 25 µm.

55. Fulcrum – Height

a. of low to medium height (Figs 14H, I, 15G, H; exemplar *S. tremula*, *S. oblonga*)

b. high to very high (Figs 14J, 15I, J, 16B; exemplar *S. longipes*, *S. vorax*, *S. grimpei*)

56. Fulcrum – Overall shape

a. slender, blade-like (Figs 14H, 15G; exemplar *S. tremula*, *S. pectinata*)

b. machete-like (Figs 14I, 15H; exemplar *S. oblonga*)

c. robust, axe-shaped to semi-circular (Figs 14J, 15I, J, 16B; exemplar *S. longipes*, *S. vorax*, *S. grimpei*)

57. Fulcrum – Shape of the distal ventral margin

a. not oblique (Figs 14H, 15G; exemplar *S. tremula*)

b. oblique (Figs 14I, J, 15H–J, 16B; exemplar *S. S. oblonga*, *S. longipes*, *S. vorax*, *S. grimpei*)

58. Fulcrum – Presence of a distinct dorsal thickening (and lamellar ventral side)

a. present (Figs 15I, 16B; exemplar *S. vorax*, *S. grimpei*)

b. absent (or very weak) (Figs 15G, H, J; exemplar *S. tremula*, *S. longipes*)

59. Hypopharynx – Width

a. small to medium, robust (Figs 14K, 15K; exemplar *S. tremula*, *S. oblonga*)

b. broad to very broad, pointed laterally / dagger-like (Figs 14L, 15L; exemplar *S. stylata*)

60. Manubrium – Shape of cauda

a. of even width (Figs 14M, 15G; exemplar *S. tremula*) or narrowing slightly distally (Figs 14N, 15H; exemplar *S. pectinata*)

b. small (Fig. 14O; exemplar *S. hutchingsi*) or large (Figs 14P, 16B; exemplar *S. grimpei*, *S. tremuloida*) knob-like thickening at the distal end

c. oar blade shaped in the distal half (Fig. 14Q; exemplar *S. oblonga*)

d. spatulate or kinked at the distal end (Fig. 14R; exemplar *S. glacialis*)

61. Manubrium – Thickness of the cauda

a. very thin, slender (Fig. 16A; exemplar *S. kitina*, *S. triophthalma*)

b. medium or robust (Figs 15G, H, J, 16B; exemplar *S. tremula*, *S. longipes*, *S. grimpei*)

#### Weighted matrix key for *Synchaeta* – detailed tables

**Table 3.** Weighted character states for habitat, (swimming) behaviour and size of species of *Synchaeta*.

Category	Habitat	Behaviour					Size
Characters	Limnic, brackish, marine	Swimming duration	Adherence to objects	Swimming motion	Foot position while swimming	Directional changes	Overall size
**Character number**	**1**	**2**	**3**	**4**	**5**	**6**	**7**
*S. arcifera*	b/c	a?	?	?	?	?	a
*S. atlantica*	c	a	?	?	?	?	a
*S. bacillifera*	c	a	?	?	?	?	b
*S. baltica*	b/c	a	a	b	a	b	b
*S. bicornis*	b	a	?	a	a?	a	*a/b*
*S. cecilia*	b/c	b	b/c	a	b	a	a
*S. cylindrica*	**d**	?	?	?	?	?	a
*S. fennica*	b/c	?	?	?	?	?	*a/b*
*S. glacialis*	c	a	?	?	?	?	a
*S. grandis*	a	a	a	c	b	b	b
*S. grimpei*	b/c	a	a	a	b	b	b
*S. gyrina*	b/c	a†	a†	b/c	b	a	*a/b*
*S. hutchingsi*	b/c	a	a	a/b	b	a	a
*S. hyperborea*	c	a	?	?	?	?	*a/b*
*S. johanseni*	c	a	?	?	?	?	b
*S. kitina*	a	b	b	a	a	a	a
*S. lakowitziana*	a	?	?	?	a?	?	b
*S. longipes*	a	a	a	a	b	a	*a/b*
*S. neapolitana*	c	?	?	?	?	?	a
*S. oblonga*	a/(b)	a†	a†	a/b	a	a	a
*S. pachypoda*	a	?	?	?	b	?	b
*S. pachypoida*	a	?	?	?	a	?	b
*S. pectinata*	a	a	a	c	b	b	b
*S. prominula*	a	?	?	?	?	?	*a/b*
*S. rousseleti*	c	a	a	?	?	?	a
*S. squamadigitata*	c	?	?	?	?	?	a
*S. stylata*	a	a	a	b	b	a	*a/b*
*S. tamara*	c	a	?	?	?	?	*a/b*
*S. tavina*	b/c	a/b‡	?	c	?	a	a
*S. tremula*	a	b	c	a	b	b	*a/b*
*S. tremuloida*	a	b	c	b	b	a	*a/b*
*S. triophthalma*	b/c	a	a	a	b	a	*a/b*
*S. verrucosa*	a	?	?	c	a	?	*a/b*
*S. vorax*	b/c	a	a	a	b	a	*a/b*

† Adheres to objects only when disturbed and then only for a short time and without any twisting movement. ‡ Adherence to objects observed by Lauterborn (1905) and Remane (1929), but not by Hood (1893).

**Table 4.** Weighted character states for the morphology of the head and neck region in species of *Synchaeta*.

Category	Apical field		Styles	Auricles		Neck	Appendages		Head
Character	Width	Elevation	Elevation	Size	Orientation	Demarcation	Presence	Location	Colour
**Character number**	**8**	**9**	**10**	**11**	**12**	**13**	**14**	**15**	**16**
*S. arcifera*	*b*	*b*	*b*?	?	*b*	*a*	b	b	?
*S. atlantica*	*b*	*a/b*	*a*	a	a	*a/b*	a	c	?
*S. bacillifera*	*b*	*c*	*b*	*c/d*	*c*	*a*	b	**a**	?
*S. baltica*	*b*	*c*	*c*	d	*c*	*c*	a	c	*c*
*S. bicornis*	*b*	*c*	*c*	d	*b/c*	*a*	b	b	*a*
*S. cecilia*	*a*	*b*	*b*	*c*	*a/b*	*a/(c?)*	a	c	?
*S. cylindrica*	*a*	*b*	*a/b*	*b*	*a/b*	*c*	a	c	?
*S. fennica*	*b*	*c*	*b*	*c/d*	*b/c*	*c*	b	b	?
*S. glacialis*	*a/b*	*c*	*a*	*b*	*b*	*c*	a	c	?
*S. grandis*	*b*	*c*	*b*	d	*c*	*a*	a	c	*a/c*
*S. grimpei*	*b*	*a*	*a*	a	a	*a*	a	c	*a-c*
*S. gyrina*	*a/b*	*a*	*b*	*b*	*a/(b)*	*b/c*	a	c	*a*
*S. hutchingsi*	*b*	*b*	*b*	*c*	*a/b*	*c*	a	c	*a/b*
*S. hyperborea*	*a*	*c*	*a*	*b*	*a/b*	*c*	a	c	?
*S. johanseni*	*b*	*c*	*b*	*c/d*	*b/c*	*c*?	a	c	*a*
*S. kitina*	*b*	*a/b*	*b*	*c*	a	*a*	a	c	*a/b*
*S. lakowitziana* †	*a/b*	*b?/c*	*b/c*	*c*	*b*	*a/c*	a	c	?
*S. longipes*	*b*	*c*	*c*	d	*c*	*a*	a	c	b
*S. neapolitana*	*b*	*b*	*b/c*	d	*a/b*	*c*	a	c	?
*S. oblonga*	*b*	*b*	*b/c*	*c*	*b*	*c*	a	c	*a/b*
*S. pachypoda*	*a/b*	*a/b*	*b*	*b/c*	*b*	*c*	a	c	?
*S. pachypoida*	*a/b*	*a*	*a*	*b*	a	*c*	a	c	?
*S. pectinata*	*b*	*c*	*a/b*	*c/d*	*c*	*a*	a	c	*a*
*S. prominula*	*a*	*b*	*b*	*b*	*a/b*	*b*	a	c	?
*S. rousseleti*	*a*	*a*	?	a	a	*a*	a	c	?
*S. squamadigitata*	*a/b*	*c*	*a*	*c*	a	*c*	a	c	?
*S. stylata*	*b*	*c*	*b*	d	*b/c*	*a*	a	c	*a*
*S. tamara*	*a*	*b*	*a*	*b*	a	*b/c*	a	c	?
*S. tavina*	*a/b*	*b*	*b*	*b*	a	*a/b*	a	c	*a*
*S. tremula*	*b*	*a*	*a*	*b*	a	*a*	a	c	*a/b*
*S. tremuloida*	*a*	*a*	*a/b*	*c*	a	*b*	a	c	*a/b*
*S. triophthalma*	*b*	*b*	*b/c*	d	*b*	*a/(c?)*	a	c	*a*
*S. verrucosa*	*b*	*c*	*a*	d	*a/b*	*c*	a	c	*a*
*S. vorax*	*b*	*c*	*c*	d	*b/c*	*c*	a	c	*a/c*

† As already noted by Hollowday (2002), this species requires further revision (preferably on living, non-preserved specimens) because of inconsistencies in the published morphological data for it, especially for the neck region and the apical field.

**Table 5.** Weighted character states for the morphology of the trunk region in species of *Synchaeta*.

Category	External morphology				Internal morphology		
Character	Shape	Anal-pseudosegment	Appendages	Longitudinal striae	Internal organs	Violet globules	Oesophagus
**Character number**	**17**	**18**	**19**	**20**	**21**	**22**	**23**
*S. arcifera*	?	?	**a**	?	a	?	?
*S. atlantica*	*c*	a	b	?	a	?	?
*S. bacillifera*	*c*	a	b	?	?	?	?
*S. baltica*	*c*	a	b	a	*a/b*	a/b	b
*S. bicornis*	*a/b*	a	b	?	*a/b*	a/b	b
*S. cecilia*	*c*	b	b	a	a	b	a
*S. cylindrica*	*b*	a	b	a	a	b	?
*S. fennica*	*a/b*	?	b	?	*a/b*	b	b
*S. glacialis*	*b*	?	b	?	a	b	a
*S. grandis*	*b*	b	b	a	*a/b*	b	b
*S. grimpei*	*a*	b	b	a	**c**	a/b	?
*S. gyrina*	*c*	a	b	a	a	b	a
*S. hutchingsi*	*a*	b	b	a	*a/b*	b	a
*S. hyperborea*	*b*	b	b	?	a	b	a?
*S. johanseni*	*c*	?	b	?	b	b	b
*S. kitina*	*a/c*	b	b	a	a	b	a
*S. lakowitziana*	*b*	?	b	?	a	b	a?
*S. longipes*	*c*	b	b	a	a/**d**	b	?
*S. neapolitana*	*a/c*	a	b	?	*a/b*	b	?
*S. oblonga*	*c*	a†	b	a	a	b	a
*S. pachypoda*	*b*	a?	b	a	a	b	a?
*S. pachypoida*	*b*	b	b	a	a	b	a?
*S. pectinata*	*a/c*	b	b	**b**	b	b	b
*S. prominula*	*c*	?	b	a	a	b	a
*S. rousseleti*	*c*	b	b	?	a	?	?
*S. squamadigitata*	*c*	b	b	a	b	b	b?
*S. stylata*	*c*	b	b	a	*a/b*	b	b
*S. tamara*	*c*	a?	b	?	a	b	?
*S. tavina*	*b*	b	b	a	a	b	a
*S. tremula*	*a*	b	b	a	a	b	a
*S. tremuloida*	*c*	a	b	a	a	b	a
*S. triophthalma*	*a*	b	b	a	(a)/b	b	a
*S. verrucosa*	*b/c*	?	b	a	b	b	a
*S. vorax*	*c*	b	b	a	a	b	b

† In contrast to Hollowday (2002), we found that *S. oblonga* exhibits a foot with only one instead of two pseudosegments. The impression of two pseudosegments being present might derive from the distinct preanal-fold that distinctly overlaps the foot, which itself is predominantly withdrawn (Wilke et al. 2018b).

**Table 6.** Weighted character states for the morphology of the foot, pedal glands, and toes in species of *Synchaeta*.

Category	Foot		Pedal glands					Toes					Other	
Character	Orientation	Shape and size	Symmetry	Arrangement	Length	Shape	Opening	Symmetry	Arrangement	Size	Separation (prox.)	Separation (dist.)	Appendages	Pseudosegments
**Character number**	**24**	**25**	**26**	**27**	**28**	**29**	**30**	**31**	**32**	**33**	**34**	**35**	**36**	**37**
*S. arcifera*	?	**a**?	b	c	a	a?	a	b	c	c	b	a	a	b
*S. atlantica*	c	**a**	b	c	c	c	?	b	c	c	b	*a/b*	a	b
*S. bacillifera*	?	*c/e*	b	c	a	c	a	b	c	b	b	b	a	b
*S. baltica*	a	*c/e*	b	c	a	c	a	b	c	a	a	b	a	b
*S. bicornis*	?	c	b	c	b	c?	a	b	c	b	b	b	a	b
*S. cecilia*	a	*b/c*	a	b	b	b	?	a	b	b	b	a	a	b
*S. cylindrica*	b	c	b	c	b	c	?	b	c	b	b	b	a	b
*S. fennica*	?	c	b	c	b	a	?	b	c	b	b	b	a	b
*S. glacialis*	b	c	b	c	b	*a/b*?	a	b	c	*b/c*	b	*a/b*	a	b
*S. grandis*	c	d	b	c	b	a	a	b	c	a	b	a	a	b
*S. grimpei*	b	e	b	c	a	e	a	b	c	a	b	b	a	b
*S. gyrina*	*b/c*	c	b	c	a	c	a	b	c	b	b	b	a	b
*S. hutchingsi*	*b/c*	b	a	b	b	*b/c*	**b**	a†	a†	b	c	c	**c**†	b
*S. hyperborea*	b	*c/e*	b	c	b	c	a	b	c	b	b	b	a	b
*S. johanseni*	?	e	b	c	a	*b/c*‡	a	b	c	*a/b*	b	b	a	b
*S. kitina*	b	*b/c*	b	c	b	b	a	b	c	*b*	b	a	a	b
*S. lakowitziana*	?	e	b	c	a	c	a	b	c	b	b	b	a	b
*S. longipes*	c	d	b	c	b	a	a	b	c	b	b	a	a	b
*S. neapolitana*	?	c	a	**a**§	b	b	a§	a|	a|	b	c	c	**b**§	a
*S. oblonga*	*b/c*	c	b	c	a¶	c	a	b	c	b	b	*a/b*	a	b
*S. pachypoda*	?	f	b	c	a	**d**	a	b	c	c	a	b	a	b
*S. pachypoida*	?	f	b	c	a	e	**c**	b	c	c	?	b	a	a
*S. pectinata*	c	c	b	c	a	a	a	b	c	*a/b*	b	a	a	b
*S. prominula*	?	c	b	c	c	b?	?	b	c	b	b	*a/b*	a	b
*S. rousseleti*	b	?	b	c	c	b	?	b	c	c	b	b	a	b
*S. squamadigitata*	?	c	b	c	b	*a/b*?	?	b	c	b	b	**d**	a	a
*S. stylata*	b	d	b	c	b	a	a	b	c	a	b	a	a	b
*S. tamara*	?	c	a	b	a/b?	b	a	a	b	b	b	?	a	a
*S. tavina*	*b/c*	c	b	c	b	c#	a	b	c	b	b	a	a	b
*S. tremula*	a	c	b	c	b	b	a	b	c	b	b	a	a	b
*S. tremuloida*	c	c	b	c	b	b	a	b	c	b	b	a	a	b
*S. triophthalma*	b	b	a	b	b	b	a	a	a	b	c	c	a	b
*S. verrucosa*	b	e	b	c	a	c	a	b	c	*b/c*	b	b	a	b
*S. vorax*	b	d	b	c	b	*a/b*	a	b	c	b	b	a	a	b

† The dorsolateral spur in *S. hutchingsi* might represent a second toe that is turned upwards because one pedal gland terminates in the spur and eggs are carried attached to it (as is the case for true toes). ‡ “b” according to the drawing by Harring (1921) and “c” according to the LM image of an individual by Jersabek et al. (2003b). § It remains to be determined whether the pedal gland of *S. neapolitana* is truly single or is highly asymmetric, with a normal, but vestigial gland that was overseen. | It remains to be determined if the dorsolateral spur in *S. neapolitana* represents a true toe (as assumed by Rousselet 1902), potentially with a pedal gland terminating in it (contra Lie-Pettersen 1905). ¶ The pedal glands are shorter than the foot (contra Hollowday 2002). The impression of the pedal glands being of foot-length might derive from the foot being partly retracted, something that is typical of *S. oblonga* (Wilke et al. 2018b). # According to the LM image of an individual by Jersabek et al. (2003b).

**Table 7.** Weighted character states for the morphology of *the sensory system in* species of *Synchaeta*.

Category	Eyes				Apical receptors		Styles	Dorsal antenna		Lateral antenna(e)			
Character	Morphology	Size	Frontal pigment granules	Streams of pigment granules	Separation	Elevation	Length of the styles	Elevation	Basal opening	Number and size	location (transversal)	location (longitudinal)	Bases of the antenna(e)
**Character number**	**38**	**39**	**40**	**41**	**42**	**43**	**44**	**45**	**46**	**47**	**48**	**49**	**50**
*S. arcifera*	b	a	?	?	c	?	*c*	?	?	c	a?	b	a
*S. atlantica*	?	?	?	?	?	?	a	?	?	?	?	?	?
*S. bacillifera*	a	a	?	?	?	?	*b*	?	?	c	?	a	a
*S. baltica*	*a/b*	b	a	a	a	b	*c*	b	b	c	b	a	a
*S. bicornis*	b	?	a	a	b	b	*c/d*	a	b	?	?	?	?
*S. cecilia*	b	a	b	b	c?	c?	*b/c*	b	?	c	a	b	b
*S. cylindrica*	b	a	?	?	?	?	*b/c*	b	?	?	?	?	?
*S. fennica*	*a/b*	a	?	?	a	e	d	b	b	c	b	a	b
*S. glacialis*	*a/b*	a	b	b	c	c	a	a	?	c	a?	a/b	b
*S. grandis*	a	a	b	b	a	b	*a/b*	a	a	c	b	a	b
*S. grimpei*	*a/b*	a	*a/b*	*a/b*	b	a	*b*	a	b	c	a	**c**	b
*S. gyrina*	b	a/(b†)	b	*a/b*	b	a	*c*	a	b	c	b	a	a
*S. hutchingsi*	a/(b)	b	*a/b*	*a/b*	c	c	*c*	a	?	a	a	b	b
*S. hyperborea*	*a/b*	a	?	?	c	c	a	a	?	c	a	a/b	b
*S. johanseni*	?	?	?	?	a	e	*c*	?	b	c	b	a	a
*S. kitina*	*b/c*	a	*a/b*	*a/b*	c	c	*c*	b	b	c	a	b	b
*S. lakowitziana*	c	a	b	b	b	*a/b*	*b/c*	b	b	c	?	a	?
*S. longipes*	*a/b*	a	b	b	a	b	d	a	b	c	b	a	b
*S. neapolitana*	*a/b*	a	b	b	c?	c?	*c*	b?	b	c	b	a	?
*S. oblonga*	*a-c*	a	*a/b*	*a/b*	b	a	*c*	a	b	c	b	a	a
*S. pachypoda*	c	a	?	?	?	?	*b/c*	?	b	c	?	a	b
*S. pachypoida*	b	a	?	?	?	?	*b/c*	?	b	c	a?	a	a
*S. pectinata*	a	a	b	b	c	**d**	*c*	a	a	c	b	a	b
*S. prominula*	b	?	?	a	b	a	*c*	?	b	c	a	b	b
*S. rousseleti*	?	?	?	?	?	?	?	?	?	?	?	?	?
*S. squamadigitata*	b	a	?	?	c	?	a	a	?	c	?	a	?
*S. stylata*	*a/b*	a	b	b	a	b	*c*	a	b	c	b	a	b
*S. tamara*	b	a	?	?	b?	a	*b/c*	?	?	**b**	**c**	b	?
*S. tavina*	*b/c*	a	?	?	b	a	*c*	b	b	c	?	a	b
*S. tremula*	b	a	b	b	b/c	a	*b/c*	b	b	c	a	b	b
*S. tremuloida*	b	a	b	b	b/c	a	*b*	b	b	c	a	b	b
*S. triophthalma*	b	a	a	a	c	c	*c*	b	b	a	a	b	b
*S. verrucosa*	c	a	*a/b*	b	?	?	*b*	b	?	c	b	a	?
*S. vorax*	b	a	*a/b*	*a/b*	a	e	d	b	b	c	b	a	b

† Pale red aggregations of pigment granules located around the darkly pigmented cerebral eyes can make the latter appear large.

**Table 8.** Weighted character states for the morphology of the trophi in species of *Synchaeta*.

Category	Ramus (“unci“)			Unci	Fulcrum				Hyp.	Cauda	
Character	Teeth presence	Shape of the teeth	Separation	Frontal hook with spur	Height	Overall shape	Distal margin	Thickening	Hypopharynx	Shape	Thickness
**Character number**	**51**	**52**	**53**	**54**	**55**	**56**	**57**	**58**	**59**	**60**	**61**
*S. arcifera*	b	e	a	a	a	a	a	b	?	*a/b*	a
*S. atlantica*	b	*c/d*	b	a	a	b	b	a?	a	b	b
*S. bacillifera*	b	d	a	b	a	a	?	?	?	?	?
*S. baltica*	b	f	a	b	a	a	a	b	*a/b*	a	b
*S. bicornis*	b	d	a	?	a	a	a	?	?	?	?
*S. cecilia* †	b	e	a	?	a	a	a	b	a	a	a
*S. cylindrica*	b	d	b	?	b	*b/c*	b	b	?	a	b
*S. fennica* ‡	a	b	b	b	b	c	b	a	?	?	?
*S. glacialis* §	b	d	b	b	*a/b*	*b/c*	b	a	a	d	b
*S. grandis*	a	*a/(b)*	*b/c*	a	a	a	b	b	b	a	b
*S. grimpei*	*a/b*	c	b	a	b	c	b	a	*a/b*	b	b
*S. gyrina*	b	d	b	b	a	b	b	b	a	*a/c*	b
*S. hutchingsi*	b	e	a	a	a	a	a	b	?	*a/b*	a
*S. hyperborea* §	b	d	b	b	a	b	b	a?	a	*a/b*	b
*S. johanseni*|	b	f	?	?	?	?	?	?	?	?	?
*S. kitina*	b	e	a	a	a	a	a	b	?	a	a
*S. lakowitziana* ¶	b	*c/d*	b	b	a	b	?	?	?	?	?
*S. longipes*	a	*a/b*	*b/c*	a	b	c	b	a	a	a	b
*S. neapolitana*#	b	e	a	a	a	a	a	b	?	?	?
*S. oblonga*	b	d	a	b	a	b	b	b	a	*a/c*	b
*S. pachypoda*	b	*c*	b	a	?	?	?	?	a	a	b
*S. pachypoida*	b	*c*	b	a	?	?	?	?	?	a	b
*S. pectinata*	a	a	c	a	a	a	a	b	*a/b*	a	b
*S. prominula*	b	d	b	?	?	?	?	?	a	*a/c*	b
*S. rousseleti*	b	*c/d*	b	a	a	b?	b	a?	a	a	b
*S. squamadigitata*	b	d	b	b	b	*b/c*	b	a	a	*b/d*	b
*S. stylata*	a	a	c	a	a	a	b	b	b	*a/d*	b
*S. tamara* §	*a/b*	*c/d*	b	b	b	*b/c*	b	a	a	a	?
*S. tavina*	b	d	?	?	b	c	b	?	?	?	?
*S. tremula*	b	d	a	b	a	a	a	b	a	a	b
*S. tremuloida*	b	d	a	b	a	a	a	b	a	b	b
*S. triophthalma*	b	e	a	a	a	a	a	b	a	a	a
*S. verrucosa* ††	*a/b*	c	b	a	b?	b	b	a	*a/b*	a	?
*S. vorax*	*a*	b	b	b	b	c	b	a	a	*a/c*	b

† Trophi according to an illustration from Arndt et al. (1990) and a LM image from Rougier et al. (2000) ‡ Trophi according to a LM image from Labuce and Strake (2017) § Trophi according to an SEM image from Friedrich and De Smet (2000) | Ramus according to a LM image of the habitus of an individual by Jersabek et al. (2003b), where the trophi were visible ¶ Trophi according to SEM and LM images from Obertegger et al. (2006) # Trophi according to LM image from Rougier et al. (2000) †† Trophi according to Jersabek et al. (2003a)

#### Weighted matrix key for *Synchaeta* – numerical list

**Table:** 

*S. arcifera*											
1b/c	2a?	?	?	?	?	7a	*8b*	*9b*	*10b*?	?	*12b*
*13a*	14b	15b	?	?	?	**19a**	?	21a	?	?	?
**25a**?	26b	27c	28a	29a?	30a	31b	32c	33c	34b	35a	36a
37b	38b	39a	?	?	42c	?	*44c*	?	?	47c	48a?
49b	50a	51b	52e	53a	54a	55a	56a	57a	58b	?	*60a/b*
61a											
*S. atlantica*											
1c	2a	?	?	?	?	7a	*8b*	*9a/b*	*10a*	11a	12a
*13a/b*	14a	15c	?	*17c*	18a	19b	?	21a	?	?	24c
**25a**	26b	27c	28c	29c	?	31b	32c	33c	34b	*35a/b*	36a
37b	?	?	?	?	?	?	44a	?	?	?	?
?	?	51b	*52c/d*	53b	54a	55a	56b	57b	58a?	59a	60b
61b											
*S. bacillifera*											
1c	2a	?	?	?	?	7b	*8b*	*9c*	*10b*	*11c/d*	*12c*
*13a*	14b	**15a**	?	*17c*	18a	19b	?	?	?	?	?
25c/e	26b	27c	28a	29c	30a	31b	32c	33b	34b	35b	36a
37b	38a	39a	?	?	?	?	*44b*	?	?	47c	?
49a	50a	51b	52d	53a	54b	55a	56a	?	?	?	?
?											
*S. baltica*											
1b/c	2a	3a	4b	5a	6b	7b	*8b*	*9c*	*10c*	11d	*12c*
*13c*	14a	15c	*16c*	*17c*	18a	19b	20a	*21a/b*	22a/b	23b	24a
*25c/e*	26b	27c	28a	29c	30a	31b	32c	33a	34a	35b	36a
37b	*38a/b*	39b	40a	41a	42a	43b	*44c*	45b	46b	47c	48b
49a	50a	51b	52f	53a	54b	55a	56a	57a	58b	*59a/b*	60a
61b											
*S. bicornis*											
1b	2a	?	4a	5a?	6a	*7a/b*	*8b*	*9c*	*10c*	11d	*12b/c*
*13a*	14b	15b	*16a*	*17a/b*	18a	19b	?	*21a/b*	22a/b	23b	?
25c	26b	27c	28b	29c?	30a	31b	32c	33b	34b	35b	36a
37b	38b	?	40a	41a	42b	43b	*44c/d*	45a	46b	?	?
?	?	51b	52d	53a	?	55a	56a	57a	?	?	?
?											
*S. cecilia*											
1b/c	2b	3b/c	4a	5b	6a	7a	*8a*	*9b*	*10b*	*11c*	*12a/b*
*13a(/c)*	14a	15c	?	*17c*	18b	19b	20a	21a	22b	23a	24a
*25b/c*	26a	27b	28b	29b	?	31a	32b	33b	34b	35a	36a
37b	38b	39a	40b	41b	42c?	43c?	*44b/c*	45b	?	47c	48a
49b	50b	51b	52e	53a	?	55a	56a	57a	58b	59a	60a
61a											
*S. cylindrica*											
**1d**	?	?	?	?	?	7a	*8a*	*9b*	*10a/b*	*11b*	*12a/b*
*13c*	14a	15c	?	*17b*	18a	19b	20a	21a	22b	?	24b
25c	26b	27c	28b	29c	?	31b	32c	33b	34b	35b	36a
37b	38b	39a	?	?	?	?	*44b/c*	45b	?	?	?
?	?	51b	52d	53b	?	55b	*56b/c*	57b	58b	?	60a
61b											
*S. fennica*											
1b/c	2?	?	?	?	?	*7a/b*	*8b*	*9c*	*10b*	*11c/d*	*12b/c*
*13c*	14b	15b	?	*17a/b*	?	19b	?	*21a/b*	22b	23b	?
25c	26b	27c	28b	29a	?	31b	32c	33b	34b	35b	36a
37b	*38a/b*	39a	?	?	42a	43e	44d	45b	46b	47c	48b
49a	50b	51a	52b	53b	54b	55b	56c	57b	58a	?	?
?											
*S. glacialis*											
1c	2a	?	?	?	?	7a	*8a/b*	*9c*	*10a*	*11b*	*12b*
*13c*	14a	15c	?	*17b*	?	19b	?	21a	22b	23a	24b
25c	26b	27c	28b	*29a/b*?	30a	31b	32c	*33b/c*	34b	*35a/b*	36a
37b	*38a/b*	39a	40b	41b	42c	43c	44a	45a	?	47c	48a?
49a/b	50b	51b	52d	53b	54b	*55a/b*	*56b/c*	57b	58a	59a	60d
61b											
*S. grandis*											
1a	2a	3a	4c	5b	6b	7b	*8b*	*9c*	*10b*	11d	*12c*
*13a*	14a	15c	*16a/c*	*17b*	18b	19b	20a	*21a/b*	22b	23b	24c
25d	26b	27c	28b	29a	30a	31b	32c	33a	34b	35a	36a
37b	38a	39a	40b	41b	42a	43b	*44a/b*	45a	46a	47c	48b
49a	50b	51a	*52a(/b)*	*53b/c*	54a	55a	56a	57b	58b	59b	60a
61b											
*S. grimpei*											
1b/c	2a	3a	4a	5b	6b	7b	*8b*	*9a*	*10a*	11a	12a
*13a*	14a	15c	*16a-c*	*17a*	18b	19b	20a	**21c**	22a/b	?	24b
25e	26b	27c	28a	29e	30a	31b	32c	33a	34b	35b	36a
37b	*38a/b*	39a	*40a/b*	*41a/b*	42b	43a	*44b*	45a	46b	47c	48a
**49c**	50b	*51a/b*	52c	53b	54a	55b	56c	57b	58a	*59a/b*	60b
61b											
*S. gyrina*											
1b/c	2a	3a	4b/c	5b	6a	*7a/b*	*8a/b*	*9a*	*10b*	*11b*	*12a(/b)*
*13b/c*	14a	15c	*16a*	*17c*	18a	19b	20a	21a	22b	23a	*24b/c*
25c	26b	27c	28a	29c	30a	31b	32c	33b	34b	35b	36a
37b	38b 39a/(b)	40b	*41a/b*	42b	43a	*44c*	45a	46b	47c	48b	
49a	50a	51b	52d	53b	54b	55a	56b	57b	58b	59a	*60a/c*
61b											
*S. hutchingsi*											
1b/c	2a	3a	4a/b	5b	6a	7a	*8b*	*9b*	*10b*	*11c*	*12a/b*
*13c*	14a	15c	*16a/b*	*17a*	18b	19b	20a	*21a/b*	22b	23a	*24b/c*
25b	26a	27b	28b	*29b/c*	**30b**	31a	32a	33b	34c	35c	**36c**
37b	38a(b)	39b	*40a/b*	*41a/b*	42c	43c	*44c*	45a	?	47a	48a
49b	50b	51b	52e	53a	54a	55a	56a	57a	58b	?	*60a/b*
61a											
*S. hyperborea*											
1c	2a	?	?	?	?	*7a/b*	*8a*	*9c*	*10a*	*11b*	*12a/b*
*13c*	14a	15c	?	*17b*	18b	19b	?	21a	22b	23a?	24b
*25c/e*	26b	27c	28b	29c	30a	31b	32c	33b	34b	35b	36a
37b	*38a/b*	39a	?	?	42c	43c	44a	45a	?	47c	48a
49a/b	50b	51b	52d	53b	54b	55a	56b	57b	58a?	59a	*60a/b*
61b											
*S. johanseni*											
1c	2a	?	?	?	?	7b	*8b*	*9c*	*10b*	*11c/d*	*12b/c*
*13c*?	14a	15c	*16a*	*17c*	?	19b	?	21b	22b	23b	?
25e	26b	27c	28a	*29b/c*	30a	31b	32c	*33a/b*	34b	35b	36a
37b	?	?	?	?	42a	43e	*44c*	?	46b	47c	48b
49a	50a	51b	52f	?	?	?	?	?	?	?	?
?											
*S. kitina*											
1a	2b	3b	4a	5a	6a	7a	*8b*	*9a/b*	*10b*	*11c*	12a
*13a*	14a	15c	*16a/b*	*17a/c*	18b	19b	20a	21a	22b	23a	24b
*25b/c*	26b	27c	28b	29b	30a	31b	32c	*33b*	34b	35a	36a
37b	*38b/c*	39a	*40a/b*	*41a/b*	42c	43c	*44c*	45b	46b	47c	48a
49b	50b	51b	52e	53a	54a	55a	56a	57a	58b	?	60a
61a											
*S. lakowitziana*											
1a	?	?	?	5a?	?	7b	*8a/b*	*9b?/c*	*10b/c*	*11c*	*12b*
*13a/c*	14a	15c	?	*17b*	?	19b	?	21a	22b	23a?	?
25e	26b	27c	28a	29c	30a	31b	32c	33b	34b	35b	36a
37b	38c	39a	40b	41b	42b	*43a/b*	*44b/c*	45b	46b	47c	?
49a	?	51b	*52c/d*	53b	54b	55a	56b	?	?	?	?
?											
*S. longipes*											
1a	2a	3a	4a	5b	6a	*7a/b*	*8b*	*9c*	*10c*	11d	*12c*
*13a*	14a	15c	16b	*17c*	18b	19b	20a	21a/d	22b	?	24c
25d	26b	27c	28b	29a	30a	31b	32c	33b	34b	35a	36a
37b	*38a/b*	39a	40b	41b	42a	43b	44d	45a	46b	47c	48b
49a	50b	51a	*52a/b*	*53b/c*	54a	55b	56c	57b	58a	59a	60a
61b											
*S. neapolitana*											
1c	?	?	?	?	?	7a	*8b*	*9b*	*10b/c*	11d	*12a/b*
*13c*	14a	15c	?	*17a/c*	18a	19b	?	*21a/b*	22b	?	?
25c	26a	**27a**	28b	29b	30a	31a	32a	33b	34c	35c	**36b**
37a	*38a/b*	39a	40b	41b	42c?	43c?	*44c*	45b?	46b	47c	48b
49a	?	51b	52e	53a	54a	55a	56a	57a	58b	?	?
?											
*S. oblonga*											
1a(/b)	2a	3a	4a/b	5a	6a	7a	*8b*	*9b*	*10b/c*	*11c*	*12b*
*13c*	14a	15c	*16a/b*	*17c*	18a	19b	20a	21a	22b	23a	*24b/c*
25c	26b	27c	28a	29c	30a	31b	32c	33b	34b	*35a/b*	36a
37b	*38a-c*	39a	*40a/b*	*41a/b*	42b	43a	*44c*	45a	46b	47c	48b
49a	50a	51b	52d	53a	54b	55a	56b	57b	58b	59a	*60a/c*
61b											
*S. pachypoda*											
1a	?	?	?	5b	?	7b	*8a/b*	*9a/b*	*10b*	*11b/c*	*12b*
*13c*	14a	15c	?	*17b*	18a?	19b	20a	21a	22b	23a?	?
25f	26b	27c	28a	**29d**	30a	31b	32c	33c	34a	35b	36a
37b	38c	39a	?	?	?	?	*44b/c*	?	46b	47c	?
49a	50b	51b	*52c*	53b	54a	?	?	?	?	59a	60a
61b											
*S. pachypoida*											
1a	?	?	?	5a	?	7b	*8a/b*	*9a*	*10a*	*11b*	12a
*13c*	14a	15c	?	*17b*	18b	19b	20a	21a	22b	23a?	?
25f	26b	27c	28a	29e	**30c**	31b	32c	33c	?	35b	36a
37a	38b	39a	?	?	?	?	*44b/c*	?	46b	47c	48a?
49a	50a	51b	*52c*	53b	54a	?	?	?	?	?	60a
61b											
*S. pectinata*											
1a	2a	3a	4c	5b	6b	7b	*8b*	*9c*	*10a/b*	*11c/d*	*12c*
*13a*	14a	15c	*16a*	*17a/c*	18b	19b	**20b**	21b	22b	23b	24c
25c	26b	27c	28a	29a	30a	31b	32c	*33a/b*	34b	35a	36a
37b	38a	39a	40b	41b	42c	**43d**	*44c*	45a	46a	47c	48b
49a	50b	51a	52a	53c	54a	55a	56a	57a	58b	*59a/b*	60a
61b											
*S. prominula*											
1a	?	?	?	?	?	*7a/b*	*8a*	*9b*	*10b*	*11b*	*12a/b*
*13b*	14a	15c	?	*17c*	?	19b	20a	21a	22b	23a	?
25c	26b	27c	28c	29b?	?	31b	32c	33b	34b	*35a/b*	36a
37b	38b	?	?	41a	42b	43a	*44c*	?	46b	47c	48a
49b	50b	51b	52d	53b	?	?	?	?	?	59a	*60a/c*
61b											
*S. rousseleti*											
1c	2a	3a	?	?	?	7a	*8a*	*9a*	?	11a	12a
*13a*	14a	15c	?	*17c*	18b	19b	?	21a	?	?	24b
?	26b	27c	28c	29b	?	31b	32c	33c	34b	35b	36a
37b	?	?	?	?	?	?	?	?	?	?	?
?	?	51b	*52c/d*	53b	54a	55a	56b?	57b	58a?	59a	60a
61b											
*S. squamadigitata*											
1c	?	?	?	?	?	7a	*8a/b*	*9c*	*10a*	*11c*	12a
*13c*	14a	15c	?	*17c*	18b	19b	20a	21b	22b	23b?	?
25c	26b	27c	28b	*29a/b*?	?	31b	32c	33b	34b	**35d**	36a
37a	38b	39a	?	?	42c	?	44a	45a	?	47c	?
49a	?	51b	52d	53b	54b	55b	*56b/c*	57b	58a	59a	*60b/d*
61b											
*S. stylata*											
1a	2a	3a	4b	5b	6a	*7a/b*	*8b*	*9c*	*10b*	11d	*12b/c*
*13a*	14a	15c	*16a*	*17c*	18b	19b	20a	*21a/b*	22b	23b	24b
25d	26b	27c	28b	29a	30a	31b	32c	33a	34b	35a	36a
37b	*38a/b*	39a	40b	41b	42a	43b	*44c*	45a	46b	47c	48b
49a	50b	51a	52a	53c	54a	55a	56a	57b	58b	59b	*60a/d*
61b											
*S. tamara*											
1c	2a	?	?	?	?	*7a/b*	*8a*	*9b*	*10a*	*11b*	12a
*13b/c*	14a	15c	?	*17c*	18a?	19b	?	21a	22b	?	?
25c	26a	27b	28a/b?	29b	30a	31a	32b	33b	34b	?	36a
37a	38b	39a	?	?	42b?	43a	*44b/c*	?	?	**47b**	**48c**
49b	?	*51a/b*	*52c/d*	53b	54b	55b	*56b/c*	57b	58a	59a	60a
?											
*S. tavina*											
1b/c	2a/b	?	4c	?	6a	7a	*8a/b*	*9b*	*10b*	*11b*	12a
*13a/b*	14a	15c	*16a*	*17b*	18b	19b	20a	21a	22b	23a	*24b/c*
25c	26b	27c	28b	29c	30a	31b	32c	33b	34b	35a	36a
37b	*38b/c*	39a	?	?	42b	43a	*44c*	45b	46b	47c	?
49a	50b	51b	52d	?	?	55b	56c	57b	?	?	?
?											
*S. tremula*											
1a	2b	3c	4a	5b	6b	*7a/b*	*8b*	*9a*	*10a*	*11b*	12a
*13a*	14a	15c	*16a/b*	*17a*	18b	19b	20a	21a	22b	23a	24a
25c	26b	27c	28b	29b	30a	31b	32c	33b	34b	35a	36a
37b	38b	39a	40b	41b	42b/c	43a	*44b/c*	45b	46b	47c	48a
49b	50b	51b	52d	53a	54b	55a	56a	57a	58b	59a	60a
61b											
*S. tremuloida*											
1a	2b	3c	4b	5b	6a	*7a/b*	*8a*	*9a*	*10a/b*	*11c*	12a
*13b*	14a	15c	*16a/b*	*17c*	18a	19b	20a	21a	22b	23a	24c
25c	26b	27c	28b	29b	30a	31b	32c	33b	34b	35a	36a
37b	38b	39a	40b	41b	42b/c	43a	*44b*	45b	46b	47c	48a
49b	50b	51b	52d	53a	54b	55a	56a	57a	58b	59a	60b
61b											
*S. triophthalma*											
1b/c	2a	3a	4a	5b	6a	*7a/b*	*8b*	*9b*	*10b/c*	11d	*12b*
*13a(/c)*	14a	15c	*16a*	*17a*	18b	19b	20a	21(a/)b	22b	23a	24b
25b	26a	27b	28b	29b	30a	31a	32a	33b	34c	35c	36a
37b	38b	39a	40a	41a	42c	43c	*44c*	45b	46b	47a	48a
49b	50b	51b	52e	53a	54a	55a	56a	57a	58b	59a	60a
61a											
*S. verrucosa*											
1a	?	?	4c	5a	?	*7a/b*	*8b*	*9c*	*10a*	11d	*12a/b*
*13c*	14a	15c	*16a*	*17b/c*	?	19b	20a	21b	22b	23a	24b
25e	26b	27c	28a	29c	30a	31b	32c	*33b/c*	34b	35b	36a
37b	38c	39a	*40a/b*	41b	?	?	*44b*	45b	?	47c	48b
49a	?	*51a/b*	52c	53b	54a	55b?	56b	57b	58a	*59a/b*	60a
?											
*S. vorax*											
1b/c	2a	3a	4a	5b	6a	*7a/b*	*8b*	*9c*	*10c*	11d	*12b/c*
*13c*	14a	15c	*16a/c*	*17c*	18b	19b	20a	21a	22b	23b	24b
25d	26b	27c	28b	*29a/b*	30a	31b	32c	33b	34b	35a	36a
37b	38b	39a	*40a/b*	41a/b	42a	43e	44d	45b	46b	47c	48b
49a	50b	*51a*	52b	53b	54b	55b	56c	57b	58a	59a	*60a/c*
61b											

## Discussion

Our weighted taxonomic matrix key constitutes the most comprehensive and comparable compilation of morphological and behavioural characters to date for the 34 species of *Synchaeta* that we consider to be valid. Through it, we hope to facilitate the reliable identification of both live as well as of preserved specimens, in part by highlighting those features that are more susceptible to the effects of preservation and, more generally, by indicating the reliability of different characters or individual character states for species identification.

In attempting to make our key as comprehensive as possible, we undertook detailed re-examinations of 14 species of *Synchaeta* (Wilke et al. 2017; Wilke et al. 2018a, Wilke et al. 2018b), supplemented by information from the literature where necessary. Nevertheless, we were restricted exclusively to literature information for 20 species of this genus, resulting in numerous cases of both missing information and uncertainty (indicated with a “?” in the tables 3–8). A pervasive problem in the literature is that many species have not been re-discovered since their initial description (e.g., *S. atlantica* and *S. rousseleti*; see Hollowday 2002) so that little information exists for them at all and that many species descriptions are extremely brief and exclusively restricted to the most obvious, diagnostic characters that discriminate the species from other known species and usually highly similar ones (e.g., *S. tremuloida*; see Pourriot 1965). Thus, it is not uncommon that important, but basic information is missing for many species such as for example the location and number of sensory antennae for *S. atlantica*, *S. rousseleti* and *S. cylindrica* (see Hollowday 2002), information that could also distinguish the species from new ones discovered in future. In addition, information is often missing or conflicting for those characters for which data are hard to obtain. For example, although the trophi are important for species identification in rotifers (De Smet 1998; Segers 2004), special skills and equipment are needed for their investigation (Telesh and Heerkloss 2002) such that they are often disregarded and so incompletely known for several species of *Synchaeta* (e.g., *S. johanseni*; see Hollowday 2002).

A further problem is that many illoricate species like those in *Synchaeta* have been described on the basis of preserved material only and, despite repeated calls not to do so (e.g., Donner 1959), fixation is commonly used in rotifer research (Labuce and Strake 2017), with its practical applications making it a necessary evil. However, in soft bodied rotifers such as *Synchaeta*, preservation is far more evil than it is necessary insofar as it causes deformations and/or distortions (Ruttner-Kolisko 1972; Koste 1978; Shiel and Koste 1993), with the consequence that species potentially include preservation-influenced characters in their respective descriptions. This, in turn, might explain why several species have only ever been found once (e.g., *S. atlantica* and *S. rousseleti*, both of which were described using preserved material). However, even more commonly reported members of *Synchaeta* were described on the basis of preserved material as well (see Hollowday 2002), including *S. lakowitziana*, which is “notoriously disputed” (Hollowday 2002; p. 103) for some aspects of its characteristic morphology (e.g., the sharp neck constriction) that are suspected to be a preparation artefact.

Altogether, these problems highlight the need for standardized and comprehensive species descriptions in *Synchaeta* as well as in rotifers more generally comprising morphological (habitus and trophi), behavioural and molecular data from both live and preserved specimens (e.g., in Wilke et al. 2017; Wilke et al. 2018a, Wilke et al. 2018b). Such an integrative approach ensures the most comprehensive data set for the respective species and facilitates an assessment of which characters are potentially affected by preservation-based deformations and to which degree. Depending on the context (e.g., ecological assessments), it will often be difficult to avoid preservation. However, knowledge of its specific effects and providing sets of characters that are robust to them will facilitate better species identification. As such, we hope that our weighted taxonomic matrix key for *Synchaeta*, both through its comprehensiveness as well as through its use of weighting to indicate character reliability and utility, will not only make species identification in *Synchaeta* easier, but will also serve as a model for future keys within rotifers.

## Footnotes

This character strongly depends on the amount of ingested food or presence of developing eggs in some species (e.g., S. gyrina; character state “a/b”).

The intensity of the colour is often variable within the species and a less intense colour can appear as colourless

The body shape is strongly influenced by the pressure of the cover glass or fixation/preservation (Koste 1978) as well as from developing eggs and stomach content.

Although the present character overlaps with the previous one, it is more specific. It should only be consulted when the state can be determined with certainty because the state “b” can be difficult to determine accurately in the case of a second rudimental gland that can be easily overseen. Otherwise, we recommend character 26 for species identification.

The length of the pedal glands is related to the foot length. Caution should be exercised with individuals that carry their foot retracted to any degree because in such situations the glands can appear longer than they really are and/or that they extend into the trunk.

The present character overlaps with the previous one but is more specific. It should only be consulted when the state can be determined with certainty because the state “b” can be difficult to determine accurately in the case of a second rudimental toe that can be easily overseen. Otherwise, we recommend character 31 for species identification.

A general re-examination is recommended here to verify if any apparent pseudosegmentation is actually an artefact caused by wrinkles (e.g., S. hyperborea; described as “wrinkled” in Friedrich and De Smet (2000), but “pseudosegmented” in Hollowday (2002)), by the insertion point of the spur (e.g., S. neapolitana) or by the preanal-fold overlapping the foot (e.g., S. oblonga; Wilke et al. 2018b).

The granules are often regarded as being frontal eyespots, but they are more likely frontal aggregations of pigmented granules of the retrocerebral organ (Wilke et al. in prep.)

The streams of pigment granules appear to be present in the ducts of the retrocerebral organ (Wilke et al. in prep.).

These states are admittedly subjective, especially in the absence of any reference point. However, for those species for which literature data only was available, it was often not possible to be more precise. As such, we have listed all states except the obvious “minute” as subjective in the associated table and highly recommend comparison with our illustrations of the exemplar species for each condition.

The toothed or edentulous plate always considered to be the uncus is actually the ramus (Wilke et al. in prep.).

See statement from character 51. This character overlaps with character 51 but is more specific. Trophi are difficult to examine in their detail, which is why the present character should only be applied when it can be answered with certainty. Otherwise, we recommend character 51 for species identification.

See statement from character 51.

The structure always described as the “frontal hook” actually represents the uncus (Wilke et al. in prep.).

## Supplementary materials

Supplementary material 1 Blank character checklists and tables Data type: Microsoft Word file Explanation note: **Table S1.** Checklist of all characters and their states. **Table S2.** Blank table for recording character states. https://binary.pensoft.net/file/324502This dataset is made available under the Open Database License (http://opendatacommons.org/licenses/odbl/1.0/). The Open Database License (ODbL) is a license agreement intended to allow users to freely share, modify, and use this Dataset while maintaining this same freedom for others, provided that the original source and author(s) are credited.Tanja Wilke, Wilko H. Ahlrichs, Olaf R.P. Bininda-Emonds
