# Supplementary material for: A weighted taxonomic matrix key for species of the rotifer genus Synchaeta (Rotifera, Monogononta, Synchaetidae)
Source: Zookeys. 2019 Aug 12;871:1–40. doi: 10.3897/zookeys.871.36435 (PMC6700057; doi:10.3897/zookeys.871.36435)
Supplement: Supplementary material 1 [file zookeys-871-001-s001.docx]

**Blank character checklists and tables**

**Table S1.** Checklist of all characters and their states.

|  | **Character & State** | | **X** | **Explanation** | **Notes** |
| --- | --- | --- | --- | --- | --- |
| **Habitat and behaviour** | **1.** Habitat | **a** |  | freshwater |  |
|  |  | **b** |  | brackish |  |
|  |  | **c** |  | marine |  |
|  |  | **d** |  | inland saline |  |
|  | **2**. Swimming duration | **a** |  | exclusively pelagic |  |
|  |  | **b** |  | long-lasting adherences to objects |  |
|  | **3.** Adherence to objects | **a** |  | absent or only when disturbed |  |
|  |  | **b** |  | long-lasting, no twisting movement |  |
|  |  | **c** |  | long-lasting, with twisting movement |  |
|  | **4.** Swimming motion | **a** |  | in a straight line |  |
|  |  | **b** |  | slightly coiled |  |
|  |  | **c** |  | distinctly coiled |  |
|  | **5.** Foot position | **a** |  | partly or fully retracted |  |
|  |  | **b** |  | not retracted |  |
|  | **6.** Directional changes | **a** |  | many sudden directional changes |  |
|  |  | **b** |  | no sudden directional changes |  |
|  | **7.** Size | **a** |  | less than 250 µm |  |
|  |  | **b** |  | more than 250 µm |  |
| **Head and neck region** | **8.** Apical field width | **a** |  | as wide as the trunk |  |
|  |  | **b** |  | wider than the trunk |  |
|  | **9.** Apical field elevation | **a** |  | slightly elevated |  |
|  |  | **b** |  | intermediate |  |
|  |  | **c** |  | strongly elevated |  |
|  | **10.** Dorsolateral styles elevation | **a** |  | not to very slightly raised |  |
|  |  | **b** |  | intermediate |  |
|  |  | **c** |  | strongly raised |  |
|  | **11.** Auricle size | **a** |  | not clearly distinct |  |
|  |  | **b** |  | small |  |
|  |  | **c** |  | medium |  |
|  |  | **d** |  | large |  |
|  | **12**. Auricle orientation | **a** |  | directed laterally |  |
|  |  | **b** |  | directed semi-caudally |  |
|  |  | **c** |  | directed caudally |  |
|  | **13.** Neck region **–**demarcation of the head and trunk regions | **a** |  | gradual transition from the head into the trunk region |  |
|  |  | **b** |  | neck is narrower than the head and trunk |  |
|  |  | **c** |  | demarcated by a sharp constriction or by distinct transversal folds |  |
|  | **14.** Saccular appendages | **a** |  | absent |  |
|  |  | **b** |  | present |  |
|  | **15.** Location of saccular appendages | **a** |  | ventral |  |
|  |  | **b** |  | dorsal |  |
|  |  | **c** |  | absent |  |
|  | **16.** Colour of head region | **a** |  | colourless / transparent |  |
|  |  | **b** |  | mastax moderately yellow or orange |  |
|  |  | **c** |  | rotatory organ yellow to orange |  |
| **Trunk** | **17.** Shape of the trunk region | **a** |  | conical |  |
|  |  | **b** |  | cylindrical |  |
|  |  | **c** |  | bell- to wineglass-shaped |  |
|  | **18.** Anal-pseudosegment | **a** |  | present |  |
|  |  | **b** |  | absent |  |
|  | **19.** Saccate appendage | **a** |  | present |  |
|  |  | **b** |  | absent |  |
|  | **20.** Longitudinal striae | **a** |  | present |  |
|  |  | **b** |  | absent |  |
|  | **21.** Location of internal organs | **a** |  | entire trunk region |  |
|  |  | **b** |  | oesophagus only structure in anterior trunk region |  |
|  |  | **c** |  | middle trunk region, cloaca ends in posterior quarter of the turnk |  |
|  |  | **d** |  | ovary and stomach each occupy separate sides of the trunk |  |
|  | **22.** Violet globules | **a** |  | present |  |
|  |  | **b** |  | absent |  |
|  | **23.** Oesophagus | **a** |  | with proventriculus |  |
|  |  | **b** |  | of equal width |  |
| **Foot, pedal glands and toes**  **Foot, pedal glands and toes** | **24.** Foot orientation | **a** |  | directed dorsally |  |
|  |  | **b** |  | coplanar |  |
|  |  | **c** |  | directed ventrally |  |
|  | **25.** Foot shape | **a** |  | minute, shorter than toes |  |
|  |  | **b** |  | tringular, medium |  |
|  |  | **c** |  | conical, short to medium |  |
|  |  | **d** |  | slender, medium to long |  |
|  |  | **e** |  | broad, long |  |
|  |  | **f** |  | cylindrical, massive |  |
|  | **26.** Symmetry of pedal glands | **a** |  | asymmetrical |  |
|  |  | **b** |  | symmetrical |  |
|  | **27.** Number of pedal glands | **a** |  | one single pedal gland |  |
|  |  | **b** |  | paired, of different shape |  |
|  |  | **c** |  | paired, of equal shape |  |
|  | **28.** Length of pedal glands | **a** |  | shorter than the foot |  |
|  |  | **b** |  | as long as the foot |  |
|  |  | **c** |  | longer than the foot |  |
|  | **29.** Shape of pedal glands | **a** |  | tubular |  |
|  |  | **b** |  | club-shaped |  |
|  |  | **c** |  | voluminous, reservoir distally |  |
|  |  | **d** |  | tubular, suspended from the trunk |  |
|  |  | **e** |  | two large voluminous sections |  |
|  | **30.** Opening of the pedal glands | **a** |  | into the tip(s) of the toe(s) |  |
|  |  | **b** |  | into a toe with the second into a spur |  |
|  |  | **c** |  | at the base of the toes |  |
|  | **31.** Toe symmetry | **a** |  | asymmetrical |  |
|  |  | **b** |  | symmetrical |  |
|  | **32.** Number and arrangement of toes | **a** |  | one single toe |  |
|  |  | **b** |  | paired toes of different shape |  |
|  |  | **c** |  | paired toes of equal shape |  |
|  | **33.** Size of the toes | **a** |  | minute |  |
|  |  | **b** |  | medium to large |  |
|  |  | **c** |  | very large |  |
|  | **34.** Proximal separation of the toes | **a** |  | bases widely separated |  |
|  |  | **b** |  | bases are in contact |  |
|  |  | **c** |  | only one toe is present |  |
|  | **35.** Distal separation of the toes | **a** |  | tips are in contact |  |
|  |  | **b** |  | tips are widely separated |  |
|  |  | **c** |  | only one toe is present |  |
|  |  | **d** |  | toes without tips, squamate |  |
|  | **36**. Additional foot appendages | **a** |  | none |  |
|  |  | **b** |  | dorsolateral spur |  |
|  |  | **c** |  | ventral spine and dorsolateral spur |  |
|  | **37.** Number of pseudosegments | **a** |  | at least two |  |
|  |  | **b** |  | only one |  |
| **Sensory system** | **38.** Morphology of the cerebral eye | **a** |  | single |  |
|  |  | **b** |  | paired, partially fused |  |
|  |  | **c** |  | paired, distinctly separated |  |
|  | **39.** Size of the cerebral eye | **a** |  | small to medium, evenly shaped |  |
|  |  | **b** |  | large, irregularily shaped |  |
|  | **40.** Aggregations of granules | **a** |  | present |  |
|  |  | **b** |  | absent |  |
|  | **41.** Streams of granules | **a** |  | present |  |
|  |  | **b** |  | absent |  |
|  | **42.** Separation of apical receptors | **a** |  | ciliary tufts not separated |  |
|  |  | **b** |  | ciliary tufts slightly separated |  |
|  |  | **c** |  | ciliary tufts distinctly separated |  |
|  | **43.** Elevation of the apical receptors | **a** |  | flat or on a slight central elevation |  |
|  |  | **b** |  | on a strong central elevation |  |
|  |  | **c** |  | on two bulges or pimples |  |
|  |  | **d** |  | on strong, paired elevations |  |
|  |  | **e** |  | on a single, tubular elevation |  |
|  | **44.** Length of the styles | **a** |  | minute |  |
|  |  | **b** |  | short |  |
|  |  | **c** |  | medium |  |
|  |  | **d** |  | long |  |
|  | **45.** Elevation of the dorsal antenna | **a** |  | none to slight elevation |  |
|  |  | **b** |  | distinct prominence |  |
|  | **46.** Basal opening of the dorsal antenna | **a** |  | slit-shaped, longer than wide |  |
|  |  | **b** |  | round |  |
|  | **47.** Number of lateral antenna(e) | **a** |  | one; right one absent |  |
|  |  | **b** |  | one, left one absent |  |
|  |  | **c** |  | paired |  |
|  | **48.** Location of the lateral antenn(e) | **a** |  | directly lateral |  |
|  |  | **b** |  | ventrolateral |  |
|  |  | **c** |  | mid-dorsal |  |
|  | **49.** Location of the lateral antenna(e) | **a** |  | posterior third of the trunk region |  |
|  |  | **b** |  | caudal-most of the trunk |  |
|  |  | **c** |  | on lateral lobes caudally to the cloaca |  |
|  | **50.** Base of the lateral antenna(e) | **a** |  | surrounded by a tubular or papillary fold |  |
|  |  | **b** |  | surrounded by a low epidermal fold |  |
|  | **51.** Ramus teeth | **a** |  | Edentulous |  |
|  |  | **b** |  | with several distinct teeth |  |
|  | **52.** Shape of ramus teeth | **a** |  | teeth absent |  |
|  |  | **b** |  | one distinct tooth, remaining serrated |  |
|  |  | **c** |  | one distinct tooth, remaining blunt |  |
|  |  | **d** |  | all teeth are distinctly incised |  |
|  |  | **e** |  | dorsal ones distinct, ventral comb-like |  |
|  |  | **f** |  | dorsal ones comb-like, ventral distinct |  |
|  | **53.** Separation of ramus teeth | **a** |  | separated by a cleft or distinctness |  |
|  |  | **b** |  | continuous row of teeth |  |
|  |  | **c** |  | no distinct teeth present |  |
|  | **54.** Spine of frontal hook | **a** |  | absent |  |
|  |  | **b** |  | present |  |
|  | **55.** Height of fulcrum | **a** |  | of low or medium height |  |
|  |  | **b** |  | hight to very high |  |
|  | **56.** Overall shape of the fulcrum | **a** |  | slender, blade-like |  |
|  |  | **b** |  | machete-like |  |
|  |  | **c** |  | robust, axe-shaped to semi-circular |  |
|  | **57.** Distal end of the fulcrum | **a** |  | not oblique |  |
|  |  | **b** |  | oblique |  |
|  | **58.** Fulcrum with dorsal thickening | **a** |  | present |  |
|  |  | **b** |  | absent |  |
|  | **59.** Hypopharynx width | **a** |  | small to medium, robust |  |
|  |  | **b** |  | broad to very broad, dagger-like |  |
|  | **60.** Shape of the cauda | **a** |  | of even width or narrowing distally |  |
|  |  | **b** |  | small or large knob-like thickening |  |
|  |  | **c** |  | oar blade shaped |  |
|  |  | **d** |  | spatulate or kinked |  |
|  | **61.** Thickness of the cauda | **a** |  | very thin, slender |  |
|  |  | **b** |  | medium or robust |  |

**Table S2:** Blank table for recording character states.

| 1 | 2 | 3 | 4 | 5 | 6 | 7 | 8 | 9 | 10 | 11 | 12 |
| --- | --- | --- | --- | --- | --- | --- | --- | --- | --- | --- | --- |
| 13 | 14 | 15 | 16 | 17 | 18 | 19 | 20 | 21 | 22 | 23 | 24 |
| 25 | 26 | 27 | 28 | 29 | 30 | 31 | 32 | 33 | 34 | 35 | 36 |
| 37 | 38 | 39 | 40 | 41 | 42 | 43 | 44 | 45 | 46 | 47 | 48 |
| 49 | 50 | 51 | 52 | 53 | 54 | 55 | 56 | 57 | 58 | 59 | 60 |
| 61 |  |  |  |  |  |  |  |  |  |  |  |
